# Smoking and BMI mediate the causal effect of education on lower back pain: observational and Mendelian randomization analyses

**DOI:** 10.3389/fendo.2024.1288170

**Published:** 2024-02-06

**Authors:** Zhangmeng Xu, Luming Qi, Huiwu Zhang, Duoduo Yu, Yushan Shi, Yaming Yu, Tianmin Zhu

**Affiliations:** ^1^ Department of Health Preservation and Rehabilitation, Chengdu University of Traditional Chinese Medicine, Chengdu, Sichuan, China; ^2^ Department of Sports Medicine, Sichuan Province Orthopedic Hospital, Chengdu, Sichuan, China; ^3^ Department of Medical Laboratory, The Affiliated Hospital of Shandong University of Traditional Chinese Medicine, Jinan, Shandong, China

**Keywords:** education, low back pain, smoking, body mass index, mediating effect, Mendelian randomization, observational study

## Abstract

**Objective:**

Low back pain (LBP) has been associated with education in previous observational studies, but the causality remains unclear. This study aims to assess the impact of education on LBP and to explore mediation by multiple lifestyle factors.

**Design:**

Univariable Mendelian randomization (MR) was performed to examine the overall effect of education on LBP. Subsequently, multivariable MR was conducted to assess both the direct effect of education on LBP and the influence of potential mediators. Indirect effects were estimated using either the coefficient product method or the difference method, and the proportion of mediation was calculated by dividing the indirect effect by the total effect. The observational study utilized data from the NHANES database collected between 1999 and 2004, and included 15,580 participants aged 20 years and above.

**Results:**

Increasing education by 4.2 years leads to a 48% reduction in the risk of LBP (OR=0.52; 95% CI: 0.46 to 0.59). Compared to individuals with less than a high school education, those with education beyond high school have a 28% lower risk of LBP (OR=0.72; 95% CI: 0.63 to 0.83). In the MR study, smoking accounts for 12.8% (95% CI: 1.04% to 20.8%) of the total effect, while BMI accounts for 5.9% (95% CI: 2.99% to 8.55%). The combined mediation effect of smoking and BMI is 27.6% (95% CI: 23.99% to 32.7%). In the NHANES study, only smoking exhibits a mediating effect, accounting for 34.3% (95% CI: 21.07% to 41.65%) of the effect, while BMI does not demonstrate a mediating role.

**Conclusions:**

Higher levels of education provide a protective effect against the risk of LBP. Additionally, implementing interventions to reduce smoking and promote weight loss among individuals with lower levels of education can also decrease this risk.

## 1 Introduction

Low back pain (LBP) is a pervasive health issue with a considerable global impact (1, 2). It is the leading cause of disability worldwide, affecting an estimated 632 million people and influencing all aspects of their lives, from occupational productivity to psychosocial well-being (3). Despite its wide-ranging effects, the etiology of LBP remains complex and multifactorial, among which the influence of lifestyle should not be overlooked (2, 4, 5).

In recent years, there has been growing interest in the social determinants of health and their role in the development and progression of chronic diseases. One such determinant, educational attainment, has been linked with a wide range of health outcomes (6–8). Education can affect health through various pathways, including healthy lifestyle, employment opportunities, and psychosocial factors, and among others (9, 10). Generally, higher levels of education are associated with better health and lower mortality (7, 11). However, the role of education in the etiology of LBP is less well understood. Although some studies have suggested that individuals with less education may have a higher prevalence of LBP (12, 13), it is unclear whether the effect of education on LBP is realized through a healthy lifestyle. It is also important to note that these studies are subject to confounding and reverse causation, making it challenging to infer a causal relationship.

In order to elucidate the causal effect of educational attainment on LBP and to understand the role of lifestyle in this relationship, we selected four unhealthy lifestyles factors (smoking, alcohol consumption, sedentary TV viewing, and high BMI) as potential mediators and investigated their complex relationships using Mendelian Randomization (MR) analysis, a method that uses genetic variation as an instrumental variable for estimating of causal effects. This approach offers a solution to address the problems of confounding and reverse causation that are common in observational studies (14, 15). MR has been increasingly used in epidemiology and has proved particularly useful in exploring causal relationships, such as education on health outcomes (16, 17).

In addition to MR, this study utilized data from the National Health and Nutrition Examination Survey (NHANES), a research program designed to assess the health and nutritional status of adults and children in the U.S (18). The NHANES data provide a valuable resource for examining the associations between education, lifestyle factors, and LBP in a representative sample of the U.S. population. With those approaches, our study aims to clarify the causal role of education in LBP and to investigate the mediating roles of smoking, alcohol consumption, leisure TV time, and BMI. By gaining insight into these relationships, we hope to contribute to the development of effective strategies for prevention and management of LBP.

## 2 Materials and methods

### 2.1 Study design

We employed two research methods to investigate the impact of education level on LBP and the mediating effects of various lifestyles. Initially, we utilized a two-sample MR approach to examine the causal relationship between education level and LBP. We also exploring potential mediators such as smoking, alcohol consumption, sedentary TV time, and BMI through multivariate Mendelian randomization (MVMR) analysis. Subsequently, to examine the robustness of the identified mediators, we conducted an observational study with data from the NHANES collected between 1999 and 2004. This study rigorously followed the guidelines outlined in Strengthening the Reporting of Observational Studies in Epidemiology using Mendelian Randomization (**Supplementary STROBE-MR Checklist**) and Strengthening the Reporting of Observational Studies in Epidemiology (**Supplementary STROBE-cross-sectional studies Checklist**).

### 2.2 Data sources

#### 2.2.1 Data for Mendelian randomization

Genetic instruments for educational attainment was obtained from the Social Science Genetic Association Consortium genome-wide association studies (GWASs) meta-analysis, which included 766,345 participants of European ancestry (19). The International Standard Classification of Education was utilized to establish the corresponding years of education for each major educational qualification. It should be noted that one standard deviation (SD) represents 4.2 years of schooling. Genetic variant data for LBP were obtained from the FinnGen consortium (https://www.finngen.fi) under the accession number: finn-b-M13_LOWBACKPAINORANDSCIATICA, which comprising 13,178 cases and 164,682 controls of European ancestry. The diagnosis of the cases was based on the World Health Organization’s International Classification of Diseases inclusion criteria (ICD-10 M54.5, ICD-9 724.2, ICD-8 728.70).

The genetic instruments for smoking phenotypes were obtained from a comprehensive meta-analysis on tobacco and alcohol consumption, which encompassed more than 30 GWASs with over 1.2 million individuals of European ancestry (20). Based on previously documented research (21), we used the smoking index to measure exposure to smoking, where larger smoking index scores represent greater exposure. Genetic variables for alcohol consumption were derived from the latest GWAS pooled analysis (22), containing genetic data from 3,383,199 individuals. From this, we extracted data for the subset of European ancestry (N=2,669,029). As indicated in most of the studies, exposure to alcohol consumption was measured as the amount of alcohol consumed per week. The genetic instruments for BMI were obtained from the Genetic Investigation of Anthropometric Traits consortium GWAS meta-analysis, which included approximately 700,000 individuals of European descent. One SD represents a difference of 4.8 kg/m^2^ (23). Genetic variables for leisure TV watching were derived from a behavioral GWAS containing 422,218 individuals. Participants will be asked: In a typical day, how many hours do you spend watching TV? The mean daily reported leisure TV watching was 2.8 h (SD 1.5h) (24).

#### 2.2.2 Data for NHANES study

The present cross-sectional study utilized NHANES data obtained from the Centers for Disease Control and Prevention for the years 1999 to 2004. The primary objective of the NHANES project is to evaluate the health and nutritional status of noninstitutionalized Americans through stratified multistage probability surveys (18). Data can be accessed via the NHANES website (http://www.cdc.gov/nchs/nhanes.htm) (accessed on August 10, 2022). Our study included individuals aged 20 years or older who had completed an interview and excluded pregnant women, as well as individuals with missing data on education, LBP, or covariates.

The potential covariates assessed in this study were based on the existing literature (4, 25–28), and included age, gender, race, marital status, household income, education level, smoking status, alcohol consumption, BMI, physical activity level, sedentary TV time as well as hypertension and diabetes mellitus. The education level is defined as the highest grade completed or the highest degree earned. LBP is considered a binary categorical variable, indicating whether LBP has occurred in the past three months. Smoking status is categorized into three groups: never smoked (fewer than 100 cigarettes in a lifetime), former smokers (more than 100 cigarettes but have quit), and current smokers (more than 100 cigarettes and still smoking). Race categories include non-Hispanic white, non-Hispanic black, Mexican American, and others. Marital status is categorized as married, living with a partner, or living alone. Family income is classified into three groups based on the poverty income ratio (PIR) (29): low (≤ 1.3), medium (1.3 to 3.5), and high (> 3.5). Drinking alcohol is defined as consuming an average of at least one alcoholic beverage per month. Physical activity levels are categorized as sedentary, moderate (at least 10 minutes of exercise resulting in light sweating or a mild to moderate increase in respiration/heart rate), and vigorous (at least 10 minutes of activity resulting in heavy sweating or increased respiration/heart rate). Sedentary TV time refers to the total amount of time spent watching TV or using a computer outside of work on a typical day. BMI is calculated using standardized techniques based on weight and height measurements. Previous diseases (hypertension and diabetes mellitus) were identified through a questionnaire by asking participants if they had ever been informed by their physician about these conditions.

### 2.3 Selection of instrumental variables

It is crucial to emphasize that the screening of instrumental variables (IV) must adhere to three fundamental assumptions (15): (1) a strong association between genetic variation and exposure factors; (2) genetic variation influencing the outcome solely through the exposure of interest; and (3) genetic variation being independent of confounders that may affect the outcome. We established a threshold of p < 5×10^-8^ to select genetic variants associated with the exposure. Additionally, single nucleotide polymorphisms (SNPs) were clumped based on the removal of linkage disequilibrium (LD) with an R^2^ > 0.001 within a 10,000 kb range using a European LD reference panel (30, 31). SNPs associated with LBP were eliminated using a threshold of p < 5×10^-6^ (32). To evaluate the strength of IVs, we computed the F-statistic, and MR analyses employed a threshold of F > 10 to prevent weak IV bias (33, 34).

### 2.4 Statistical analysis

We initially conducted two-sample univariable MR (UVMR) to estimate the total effect of education on LBP (γ), as well as the effects of education on each potential mediator (α). Subsequently, we treated these potential mediators as exposures and extracted their respective IVs, while removing SNPs that overlapped with education. Then performed UVMR again to examine the effects of each potential mediator on LBP. Finally, we incorporate the potential mediators identified by UVMR, along with education, into MVMR to construct various models. These models were used to investigate the independent effects of these mediators on LBP (β) and the direct effects of education on LBP (γ*). Our primary analytical method employed inverse variance weighting (IVW) modeling, which is statistically more effective when all IVs are valid (35).

In the NHANES study, data were weighted using interview weights. For statistical description, categorical variables were expressed as proportions (%), while continuous variables were described using either the mean with SD or median with interquartile range (IQR). To compare differences between groups within the complex survey sample, we employed the Wilcoxon rank-sum test and the chi-squared test, the latter with Rao & Scott’s second-order correction (36, 37). Logistic regression, adjusted for the complex survey design, was used to determine odds ratios (ORs) and 95% confidence intervals (95%CIs) for assessing the associations between covariates and LBP. We also used an adjusted linear regression model for continuous outcomes such as BMI. Various models were developed to analyze the association between education level and LBP. Subgroup analyses, based on potential mediating factors including smoking status (never vs. former or current smokers), alcohol consumption (no vs. yes), BMI (<25 vs. ≥25 kg/m^2^), and TV watching time (<3 vs. ≥3 hours per day), were conducted. Interactions between subgroups and education level were examined using likelihood ratio tests.

Our analyses were conducted using the “TwoSampleMR” and “MendelianRandomization” packages for MR, “mice” for imputation, and “survey” for weighting in R software (version 4.3.1, R Foundation for Statistical Computing, Vienna, Austria, http://www.R-project.org). We performed a total of nine UVMR analyses and four MVMR analyses, with statistical significance determined using the Bonferroni correction: p<0.05/13 (approximately 3.85×10^−3^).

### 2.5 Mediating effects analysis

To decompose the overall effect (γ) of education on LBP, we considered (i) the direct effect (γ*) of education on LBP after adjusting for each mediator, and (ii) the indirect effects of education through each mediator. The indirect effect of each mediator was calculated using the product method; for instance, the indirect effect of education through smoking on LBP was determined by multiplying the effect of education on smoking (α) with the effect of smoking on LBP (β) (38). To derive the joint indirect effect of smoking and BMI, we employed a differences method (γ-γ*), where γ* is the direct effect after adjusting for both smoking and BMI (15). For all mediators, the proportion of mediated effects was quantified by dividing their respective indirect effects by the total effect (39).

In the NHANES study, education level was reclassified into two categories: high school level or below, and above high school level. Similarly, smoking status was categorized as never smoked, former or current smoker. Multivariable logistic regression was employed to investigate the association between education and mediators (α’), while also examining the impact of mediators on LBP (β’).

### 2.6 Sensitivity analysis

In MR analysis, we used MR-Egger, weighted median, weighted mode, and mv-lasso (40) (applied in MVMR) as complementary methods to test the robustness of IVW results. The consistency of the results from these various methods should provide greater robustness against bias from horizontal pleiotropy. Heterogeneity was assessed using Cochran’s Q statistic in both IVW and MR-Egger (41). To address pleiotropy, we performed hypothesis testing on the MR-Egger intercept, removed outliers SNPs with MRPRESSO package (42), and visualized the results using leave-one-out analysis, forest plots, funnel plots, and scatter plots. Following the approach outlined by Burgess (43), we conducted analyses on each overlapping dataset to assess bias and type 1 error rates (https://sb452.shinyapps.io/overlap/). In addition, we conducted secondary analyses using different datasets to verify the robustness of our findings.

In the NHANES study, multiple imputations were conducted using the “mice” package with chained equations to impute missing values for covariates (44), excluding education level, LBP, smoking status, and BMI. Five datasets were generated in total. We analyzed each dataset separately and applied the Rubin’s rules to combine their estimates and variances, resulting in a final outcome (45). Additionally, we excluded participants with BMI outside the range of 18.5 to 40 kg/m^2^ and reanalyzed to assess the robustness of the results.

## 3 Results

### 3.1 Instrumental variables and demographic characteristics

We acquired summary data on the association between SNPs and phenotypes from GWAS for each respective phenotype (**Supplementary Table S1**). A total of 317 SNPs were selected as IVs for education with a strong instrument indicated by an F-statistic of 19.6. For smoking index, alcohol consumption, BMI, and leisure TV, we extracted 123 SNPs (F=17.8), 98 SNPs (F=16.9), 521 SNPs (F=29.3), and 148 SNPs (F=17.1) respectively, ensuring the absence of weak IVs.

**Figure 1:**
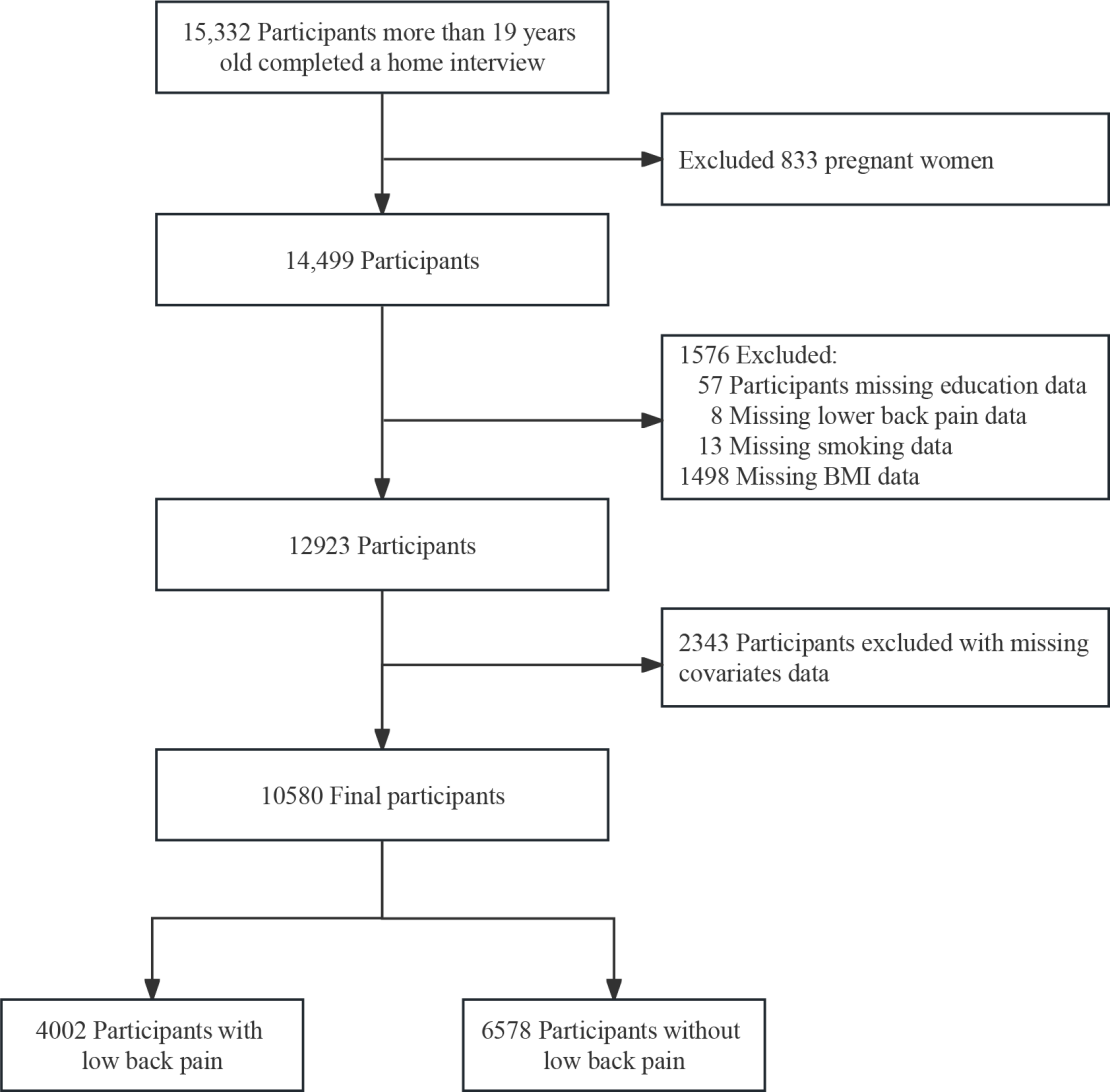
The NHANES study’s flow diagram.

A total of 15,332 participants over the age of 19 completed interviews in the NHANES study conducted between 1999 and 2004. After excluding pregnant women (n=833) and individuals with missing data on educational attainment (n=57), LBP (n=8), smoking status (n=13), BMI (n=1498) and other covariates (n=2343), a total of 10,580 participants were included. The detailed process of inclusion and exclusion is illustrated in **Figure 1**.

The **Supplementary Table S2** presents the weighted basic characteristics of both excluded and included participants. Overall, compared with the excluded group, the included group had a higher proportion of males, non-Hispanic whites, more than high school, married, high-income, consume alcohol and engage in exercise. **Table 1** provides a baseline characterization of participants categorized by education level, with 39% of participants suffering from LBP. The mean age was 46.0 (16.4) years, and females accounted for 50%. A higher level of education is associated with a higher rate of alcohol consumption, increased physical activity, less smoking, fewer diagnosis of hypertension/diabetes, less time watching TV and a lower prevalence of LBP.

**Table 1:** Weighted population characteristics by categories of education level.

Characteristic	Overall, N^1 = ^10580	Less than high school, N^1 = ^3302	High school diploma, N^1 = ^2520	More than high school, N^1 = ^4758	*P* Value^2^
Age (y), Mean ± SD	46.0 ± 16.4	49.2 ± 18.4	46.3 ± 16.9	44.7 ± 15.2	<0.001
Gender, n (%)					0.3
Male	5,401 (50%)	1,743 (51%)	1,269 (50%)	2,389 (49%)	
Female	5,179 (50%)	1,559 (49%)	1,251 (50%)	2,369 (51%)	
BMI (kg/m2), Mean ± SD	28.1 ± 6.3	28.5 ± 6.5	28.6 ± 6.4	27.8 ± 6.2	<0.001
Race, n (%)					<0.001
Non-Hispanic white	5,549 (74%)	930 (51%)	1,564 (78%)	3,055 (80%)	
Non-Hispanic black	2,014 (10%)	721 (17%)	453 (9.4%)	840 (8.4%)	
Mexican American	2,310 (7.1%)	1,413 (20%)	369 (5.4%)	528 (3.5%)	
Others	707 (8.8%)	238 (12%)	134 (6.9%)	335 (8.5%)	
Marital status, n (%)					<0.001
Married or living with a partner	6,561 (65%)	1,967 (60%)	1,522 (64%)	3,072 (68%)	
Living alone	4,019 (35%)	1,335 (40%)	998 (36%)	1,686 (32%)	
Family income, n (%)					<0.001
Low	2,973 (21%)	1,653 (46%)	653 (22%)	667 (12%)	
Medium	4,101 (36%)	1,297 (39%)	1,166 (44%)	1,638 (31%)	
High	3,506 (43%)	352 (15%)	701 (34%)	2,453 (57%)	
Alcohol consumption, n (%)					<0.001
No	3,247 (27%)	1,218 (36%)	802 (28%)	1,227 (23%)	
Yes	7,333 (73%)	2,084 (64%)	1,718 (72%)	3,531 (77%)	
Activity, n (%)					<0.001
Sedentary	4,563 (35%)	2,083 (59%)	1,104 (40%)	1,376 (25%)	
Moderate	2,977 (30%)	714 (23%)	763 (31%)	1,500 (31%)	
Vigorous	3,040 (35%)	505 (19%)	653 (29%)	1,882 (44%)	
Smoke, n (%)					<0.001
Never	5,291 (49%)	1,514 (41%)	1,159 (43%)	2,618 (55%)	
Former	2,890 (25%)	919 (25%)	678 (25%)	1,293 (26%)	
Current	2,399 (25%)	869 (35%)	683 (32%)	847 (19%)	
Hypertension or Diabetes, n (%)					<0.001
No	7,216 (74%)	2,038 (67%)	1,707 (73%)	3,471 (78%)	
Yes	3,364 (26%)	1,264 (33%)	813 (27%)	1,287 (22%)	
Watching TV time (hours per day), Median (IQR)	3.0 (1.0, 5.0)	3.0(2.0, 6.0)	3.0 (1.0, 5.0)	2.0(1.0, 4.0)	<0.001
Low back pain, n (%)					<0.001
No	6,578 (61%)	1,986 (54%)	1,483 (56%)	3,109 (65%)	
Yes	4,002 (39%)	1,316 (46%)	1,037 (44%)	1,649 (35%)	

^1^ Unweighted number, the proportions and means are weighted.

^2^ Wilcoxon rank-sum test for complex survey samples; chi-squared test with Rao & Scott’s second-order correction.

BMI, body mass index; SD, standard deviation; IQR, interquartile range.

### 3.2 The effects of education and potential mediators on LBP

**Figure 2:**
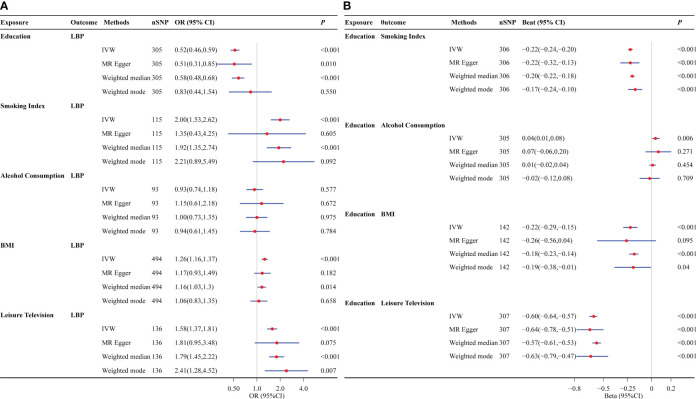
Univariate Mendelian randomization analysis. **(A)** The effects of education and potential mediators on lower back pain. **(B)** The effects of education on potential mediators. Potential mediators include smoking index, alcohol consumption, BMI, and leisure TV time. LBP, low back pain; BMI, body mass index; OR, odds ratio; IVW, inverse variance weighting.

In the UVMR analysis, per one SD increase in education was associated with a 48% decrease in the odds of LBP, with an OR of 0.52 (95% CI: 0.46 to 0.59). For the four potential mediators analyzed, alcohol consumption was not associated with LBP (OR=0.93; 95% CI: 0.74 to 1.18). The unadjusted associations of smoking, BMI, and leisure TV with LBP were observed with ORs of 2.00 (95%CI: 1.53 to 2.62), 1.26 (95%CI: 1.16 to 1.37), and 1.58 (95%CI: 1.37 to 1.81), respectively. The effects of education on smoking were (β= -0.22; 95%CI: -0.24 to -0.20), as well as on alcohol consumption (β= 0.04; 95%CI: 0.01 to 0.08), BMI (β= -0.22; 95%CI: -0.29 to -0.15), and leisure TV time (β= -0.60; 95%CI: -0.64 to -0.57). These findings are illustrated in **Figure 2**.

In the NHANES study, we initially examined the association of each covariate and LBP using univariate logistic regression (**Supplementary Table S3**). The results revealed that gender, race, BMI, family income, smoking status, physical activity level, watching TV time, as well as hypertension or diabetes mellitus were associated with the prevalence of LBP. However, no association was observed between alcohol consumption and LBP, which is consistent with the result obtained from MR analysis.

**Figure 3:**
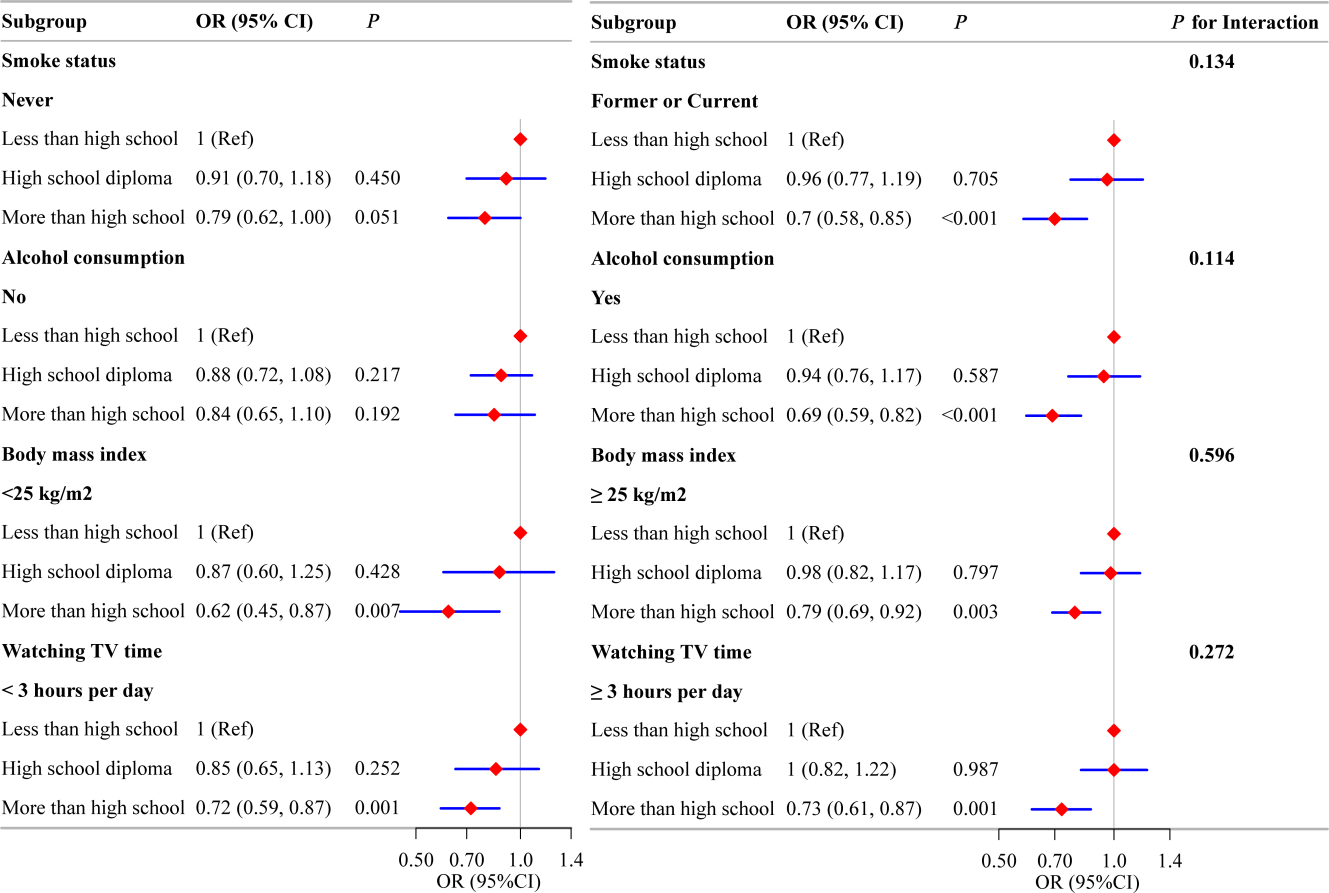
The association between education level and low back pain in subgroups. Each stratification factor was adjusted for all other variables (age, sex, marital status, race, household income, smoking status, physical activity, alcohol consumption, hypertension or diabetes mellitus, body mass index, and time spent watching television) except for the stratification component itself. LBP, low back pain; OR, odds ratio.

In the multifactorial analysis, we constructed multiple models to adjust for confounding variables. Although age, marital status, and alcohol consumption did not show an association with LBP in the univariate analysis, these factors were included in the models based on the existing literature (46, 47). The findings consistently demonstrated an inverse association between education level and the prevalence of LBP. After adjusting for all confounders, individuals with education beyond high school had reduced odds of LBP (OR=0.72; 95%CI: 0.63 to 0.83), compared to those with less than a high school education, as detailed in **Table 2**. The results of the subgroup analysis showed that the association between education level and LBP remained consistent across all subgroups. Furthermore, no interaction effects were observed between education level and the potential mediating factors, as illustrated in **Figure 3**.

**Table 2:** The weighted Association between education level and low back pain.

Education level	Crude		Model-1		Model-2	
	OR (95% CI)	p-value	OR (95% CI)	p-value	OR (95% CI)	p-value
Less than high school	1(Ref)		1(Ref)		1(Ref)	
High school diploma	0.93 (0.80, 1.08)	0.327	0.91 (0.77, 1.08)	0.269	0.93 (0.79, 1.10)	0.364
More than high school	0.63 (0.57, 0.71)	<0.001	0.66 (0.58, 0.76)	<0.001	0.7 (0.61, 0.80)	<0.001
Trend.test		<0.001		<0.001		<0.001
Education level	Model-3		Model-4		Model-5	
	OR (95% CI)	p-value	OR (95% CI)	p-value	OR (95% CI)	p-value
Less than high school	1(Ref)		1(Ref)		1(Ref)	
High school diploma	0.94 (0.79, 1.11)	0.437	0.91 (0.78, 1.08)	0.614	0.93 (0.79, 1.09)	0.337
More than high school	0.73 (0.63, 0.83)	<0.001	0.7 (0.61, 0.79)	<0.001	0.72 (0.63, 0.83)	<0.001
Trend.test		0.001		<0.001		<0.001

OR, odds ratio; Ref, reference. Crude, unadjusted for confounders. Model-1, adjusts for age, gender, race, marital status, family income. Model-2, adjusts for model-1 + watching TV time, alcohol consumption, physical activity, and hypertension or diabetes status. Model-3, adjusts for model-2 + smoking status. Model-4, adjusts for model-2 + body mass index. Model-5, adjusts for model-2 + smoking status and body mass index.

### 3.3 Mediating effect

**Figure 4:**
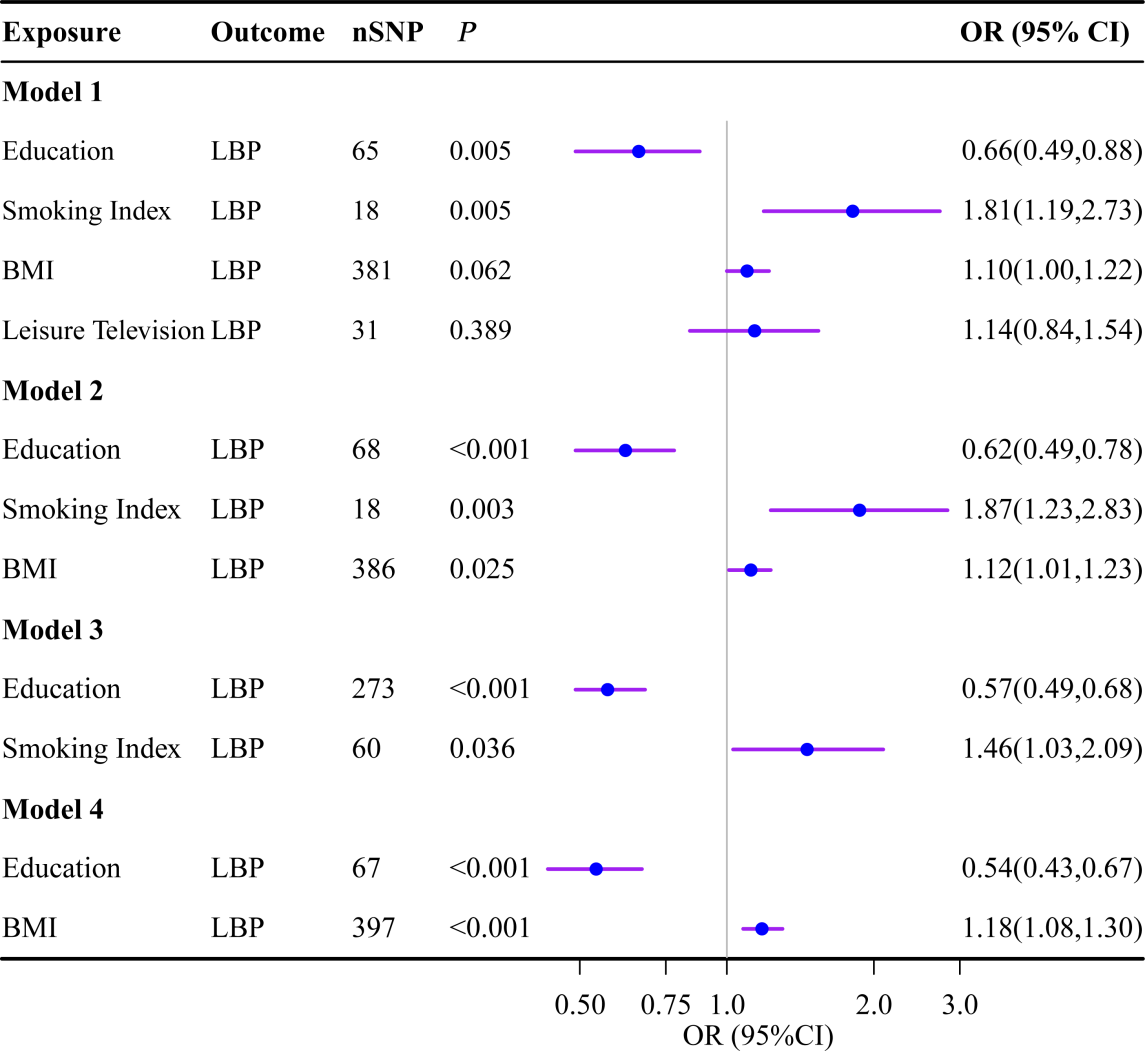
Multivariate Mendelian randomization analysis. The OR is derived from the method of inverse variance weighting. BMI, body mass index; LBP, low back pain; OR, odds ratio; SNP, single nucleotide polymorphism.

In the UVMR analysis, alcohol consumption showed no effect on LBP and was consequently excluded from subsequent MVMR analysis. In screening potential mediators, we constructed four models that individually adjusted for different mediators, as depicted in **Figure 4**. The results consistently indicated a direct effect of education on LBP with no evidence of complete mediation. After adjusting for education, BMI, and smoking index in model-1, the OR for leisure TV time became insignificant (OR=1.14; 95%CI: 0.84 to 1.54), thus excluding it from further analysis. The adjusted causal effects of smoking and BMI on LBP were OR=1.46 (95% CI: 1.03 to 2.09) and OR=1.18 (95% CI: 1.08 to 1.30), respectively.

In the NHANES study, after adjusting for other covariates, education level had an inverse association with smoking status (OR=0.62; 95%CI: 0.56 to 0.68); however, it no longer demonstrated an association with BMI (β= -0.16, 95%CI: -0.47 to 0.15). Additionally, both smoking status and BMI were independently associated with the occurrence of LBP, with ORs of 1.24 (95%CI: 1.10 to 1.40) and 1.02 (95%CI: 1.01 to 1.03), respectively, as shown in **Supplementary Table S4**.

**Figure 5:**
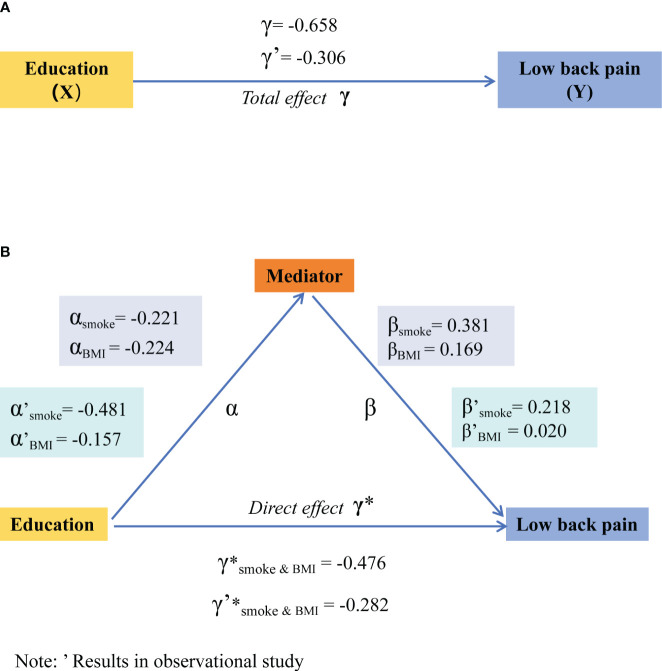
Total effect of education on low back pain **(A)** and mediating effects model for smoking and BMI **(B)**. The efficiency values in Mendelian randomization are derived from the method of inverse variance weighting, while in the NHANES study, they are obtained through weighted multivariable logistic regression. BMI, body mass index; NHANES, National Health and Nutrition Examination Survey.

In the MR analysis, the total effect (γ, as shown in **Figure 5**) of education level on LBP was -0.658 (95% CI: -0.79 to -0.53), and in the NHANES study, it was -0.306 (95% CI: -0.39 to -0.22). The direct effect (γ*, as shown in **Figure 5**) was -0.476 (95% CI: -0.71 to -0.24) in the MR analysis and -0.282 (95% CI: -0.37 to -0.20) in the NHANES study, respectively. The mediation analysis revealed that smoking accounted for 12.8% (95% CI: 10.4 to 20.8) of the total effect in the MR study, while BMI accounted for 5.90% (95% CI: 2.99 to 8.55), and their combined mediated proportion was 27.6% (95% CI: 23.99 to 32.7). In contrast, in the NHANES study, only smoking showed a mediating effect with a proportion of 34.3% (95% CI: 21.07 to 41.65), whereas BMI did not act as a mediator, as detailed in **Table 3**.

**Table 3:** Mediating effects of smoking and body mass index.

Mediator	Methods	Mediation effect	*p*-value	Mediation proportion (%)
		β (95% CI)		P (95% CI)
Smoking	MR	-0.084 (-0.164, -0.005)	0.039	12.8 (1.04, 20.8)
BMI	MR	-0.039 (-0.067, -0.015)	0.006	5.90 (2.99, 8.55)
Smoking & BMI	MR	-0.181 (-0.189, -0.173)	<0.001	27.6 (23.99, 32.7)
Smoking	NHANES study	-0.105 (-0.164, -0.046)	<0.001	34.3 (21.07, 41.65)
BMI	NHANES study	-0.003 (-0.009, 0.003)	0.307	No mediating effect

BMI, body mass index. MR, Mendelian randomization. NHANES, National Health and Nutrition Examination Survey; P, proportion.

### 3.4 Sensitivity analysis

In the MR analysis, we observed heterogeneity among the studies (**Supplementary Table S5** and **Supplementary Figures S1–4**); however, no pleiotropy was detected (**Supplementary Table S6** and **Supplementary Figures S1–4**). To explore potential SNPs exerting substantial effects on the outcomes, we reanalyzed the data after removing outlier SNPs and generated scatter plots (**Supplementary Figure S5**), funnel plots (**Supplementary Figure S6**), leave-one-out plots (**Supplementary Figure S7**), and forest plots (**Supplementary Figure S8**). Despite conducting this comprehensive analysis, none of the SNPs exhibited an impact on the results.

Furthermore, upon implementing the MR-Egger, weighted median, weighted mode, and mv-lasso (applied in the MVMR) methods, we observed an expansion of the 95%CI, resulting in a loss of statistical significance for certain findings. However, it is important to note that the causal direction remained consistent with the IVW method (**Figure 2** and **Supplementary Figures S1–4**). Lastly, we conducted a reassessment of the causal relationship between educational attainment, potential mediating variables, and LBP using an alternative dataset and obtained consistent results (**Supplementary Table S7**). However, in the analysis of sample overlap, we observed that high overlap rates introduce bias and increase the likelihood of Type I errors in the causal effect between education and smoking, alcohol consumption, and leisure TV time (**Supplementary Table S8**).

Within our NHANES study, we employed two distinct strategies: a multiple imputation approach and the exclusion of extreme BMI values. The findings derived from both methods were consistent with our initially presented results, reinforcing the hypothesis that an elevated level of education is inversely associated with the prevalence of LBP (**Supplementary Tables S9–10**).

## 4 Discussion

Our findings reveal an inverse association between education level and the prevalence of LBP, as evidenced by both the MR analysis and NHANES database. Smoking was identified as a crucial mediator in the causal relationship between education and LBP, whereas BMI only exhibited mediating effects in the MR study.

Our findings align with previous research on the impact of education on LBP. For example, a prospective study demonstrated a reduction in disability due to back pain with increasing levels of education (48). Additionally, a meta-analysis of 64 studies revealed a higher likelihood of experiencing disabling back pain among individuals with lower educational attainment (49).

Mediation analysis revealed smoking and BMI as mediating variables in the causal pathway from education to LBP, which aligns with previous research indicating that lower education levels are associated with higher rates of smoking and obesity (50), both of which can elevate the risk of LBP (4, 27). However, our NHANES database study found only smoking, not BMI, to be a mediator. This finding appears inconsistent with some prior studies (51). We have tried to explore possible reasons for this discrepancy as follows. One possibility is that in the NHANES study, education is included as a categorical variable in the model, which may obscure the relationship with BMI. Another possibility is that there may exist a non-linear relationship between BMI and education, which would not be captured by standard multivariable regression analysis (52). Furthermore, it is important to take into account factors such as diet and levels of physical activity that may influence the relationship between BMI and education (53). In summary, while our analysis did not find a significant correlation between education and BMI, this does not conclusively disprove a potential relationship. The relationship between BMI and education could be complex and influenced by various factors. Future research employing advanced statistical methods and accounting for potential confounders and interaction effects might provide further insights into this complex topic.

Another intriguing aspect of our study is the finding that smoking and BMI together mediate 27.6% (95% CI: 23.99 to 32.7) of the effect of education on LBP, leaving approximately 3/4 of the effect unaccounted for. This further highlights the complex relationship between education and LBP. It is well known that individuals with higher education attainment are more likely to engage in cognitive work as opposed to physical labor. Furthermore, they often report greater job satisfaction and enjoy better access to quality healthcare resources (54, 55), which previous research has identified as significant determinants of chronic LBP (56). Therefore, the potential role of these non-lifestyle factors in mediating the impact of education on LBP warrants further investigation.

Our findings highlight the importance of targeting smoking cessation in the prevention and management of LBP, especially among individuals with lower educational attainment. Smoking has been implicated in impairing blood flow, leading to reduced oxygen and nutrient supply to spinal tissues, which may promote the development of LBP (57). Furthermore, nicotine may increase pain sensitivity, potentially intensifying LBP symptoms (58). Therefore, interventions that reduce smoking prevalence could significantly alleviate the LBP burden. Although BMI was not identified as a mediator between education and LBP in the NHANES study, obesity remains a notable risk factor for LBP. It is hypothesized that obesity, especially abdominal obesity, exerts additional mechanical stress on the lower back, resulting in pain (59). In addition, adipose tissue can secrete pro-inflammatory cytokines, which could contribute to LBP (60, 61). Accordingly, weight management may still offer benefits for LBP prevention and treatment.

Our study still has several limitations. First, the smoking index used in MR was derived from a combination of various smoking-related indicators, not all of which were available in the NHANES database. As a result, we could only classify smoking status into two or three categories in the NHANES study, a categorization that is dimensionally different from that used in MR research. This may lead to some inconsistencies between the findings of the two methods. Second, despite excluding SNPs associated with known confounders in MR and adjusting for them in the NHANES study, residual confounding effects due to unmeasured or unknown factors cannot be completely ruled out. Third, in MR studies, partial sample overlap between education and smoking, alcohol consumption, and leisure TV time introduces bias and increases the probability of Type I errors. Further research is still needed to confirm the causal relationship among them. Additionally, the categorization of education into two groups for calculating the direct and indirect effects on LBP during the mediation analysis may result in a loss of granularity in the measurement of the exposure. These limitations emphasizes the need for further research to refine the evidence in this area.

## 5 Conclusions

In conclusion, education can reduce the prevalence of LBP, partly through its effects on smoking cessation and weight management. This implies the necessity of comprehensive strategies to prevent and manage LBP, which not only encompass direct interventions like pain management and physical therapy but also emphasize the importance of education and promoting healthy lifestyles.

## Data availability statement

The original contributions presented in the study are included in the article/**Supplementary Material**. Further inquiries can be directed to the corresponding authors.

## Ethics statement

Ethical approval was not required for the study involving humans in accordance with the local legislation and institutional requirements. Written informed consent to participate in this study was not required from the participants or the participants’ legal guardians/next of kin in accordance with the national legislation and the institutional requirements.

## Author contributions

ZX: Conceptualization, Methodology, Writing – original draft. LQ: Methodology, Resources, Software, Writing – original draft. HZ: Data curation, Investigation, Writing – review & editing. DY: Data curation, Methodology, Software, Writing – review & editing. YS: Validation, Visualization, Writing – review & editing. YY: Conceptualization, Methodology, Supervision, Writing – review & editing. TZ: Conceptualization, Project administration, Resources, Supervision, Writing – review & editing.

## References

[1] GBD 2019 Diseases and Injuries Collaborators. Global burden of 369 diseases and injuries in 204 countries and territories, 1990-2019: a systematic analysis for the Global Burden of Disease Study 2019. Lancet Lond Engl (2020) 396:1204–22. doi: 10.1016/S0140-6736(20)30925-9 PMC7567026 33069326

[2] Clark S Horton R. Low back pain: a major global challenge. Lancet Lond Engl (2018) 391:2302. doi: 10.1016/S0140-6736(18)30725-6 29573869

[3] Hoy D March L Brooks P Blyth F Woolf A Bain C. The global burden of low back pain: estimates from the Global Burden of Disease 2010 study. Ann Rheum Dis (2014) 73:968–74. doi: 10.1136/annrheumdis-2013-204428 24665116

[4] Knezevic NN Candido KD Vlaeyen JWS Van Zundert J Cohen SP. Low back pain. Lancet Lond Engl (2021) 398:78–92. doi: 10.1016/S0140-6736(21)00733-9 34115979

[5] Buchbinder R van Tulder M Öberg B Costa LM Woolf A Schoene M. Low back pain: a call for action. Lancet Lond Engl (2018) 391:2384–8. doi: 10.1016/S0140-6736(18)30488-4 29573871

[6] Che B Shen S Zhu Z Wang A Xu T Peng Y. Education level and long-term mortality, recurrent stroke, and cardiovascular events in patients with ischemic stroke. J Am Heart Assoc (2020) 9:e016671. doi: 10.1161/JAHA.120.016671 32779506 PMC7660803

[7] Herndon JE Kornblith AB Holland JC Paskett ED. Patient education level as a predictor of survival in lung cancer clinical trials. J Clin Oncol Off J Am Soc Clin Oncol (2008) 26:4116–23. doi: 10.1200/JCO.2008.16.7460 PMC2654374 18757325

[8] Davies NM Howe LD Bann D. Educational attainment and health. BMJ (2023) 382:1602. doi: 10.1136/bmj.p1602 37479240

[9] Cohen AK Syme SL. Education: a missed opportunity for public health intervention. Am J Public Health (2013) 103:997–1001. doi: 10.2105/AJPH.2012.300993 23597373 PMC3698749

[10] Davies NM Dickson M Davey Smith G Windmeijer F van den Berg GJ. The causal effects of education on adult health, mortality and income: evidence from Mendelian randomization and the raising of the school leaving age. Int J Epidemiol (2023) 52:1878–1886. doi: 10.1093/ije/dyad104 PMC10749779 37463867

[11] Montez JK Hummer RA Hayward MD. Educational attainment and adult mortality in the United States: a systematic analysis of functional form. Demography (2012) 49:315–36. doi: 10.1007/s13524-011-0082-8 PMC3290920 22246797

[12] Lourenço S Correia S Alves L Carnide F Silva S Lucas R. Intergenerational educational trajectories and lower back pain in young women and men. Acta Reumatol Port (2017) 42:73–81. 27182795

[13] Parreira P Maher CG Steffens D Hancock MJ Ferreira ML. Risk factors for low back pain and sciatica: an umbrella review. Spine J Off J North Am Spine Soc (2018) 18:1715–21. doi: 10.1016/j.spinee.2018.05.018 29792997

[14] Sekula P Del Greco MF Pattaro C Köttgen A. Mendelian randomization as an approach to assess causality using observational data. J Am Soc Nephrol JASN (2016) 27:3253–65. doi: 10.1681/ASN.2016010098 PMC5084898 27486138

[15] Carter AR Sanderson E Hammerton G Richmond RC Davey Smith G Heron J. Mendelian randomisation for mediation analysis: current methods and challenges for implementation. Eur J Epidemiol (2021) 36:465–78. doi: 10.1007/s10654-021-00757-1 PMC8159796 33961203

[16] Wang Y Ye C Kong L Zheng J Xu M Xu Y. Independent associations of education, intelligence, and cognition with hypertension and the mediating effects of cardiometabolic risk factors: a mendelian randomization study. Hypertens Dallas Tex 1979 (2023) 80:192–203. doi: 10.1161/HYPERTENSIONAHA.122.20286 PMC9722390 36353998

[17] Seyedsalehi A Warrier V Bethlehem RAI Perry BI Burgess S Murray GK. Educational attainment, structural brain reserve and Alzheimer’s disease: a Mendelian randomization analysis. Brain J Neurol (2023) 146:2059–74. doi: 10.1093/brain/awac392 PMC10151197 36310536

[18] Zipf G Chiappa M Porter KS Ostchega Y Lewis BG Dostal J. National health and nutrition examination survey: plan and operations, 1999-2010. Vital Health Stat Ser 1 Programs Collect Proced (2013) 56:1–37. 25078429

[19] Lee JJ Wedow R Okbay A Kong E Maghzian O Zacher M. Gene discovery and polygenic prediction from a genome-wide association study of educational attainment in 1.1 million individuals. Nat Genet (2018) 50:1112–21. doi: 10.1038/s41588-018-0147-3 PMC6393768 30038396

[20] Liu M Jiang Y Wedow R Li Y Brazel DM Chen F. Association studies of up to 1.2 million individuals yield new insights into the genetic etiology of tobacco and alcohol use. Nat Genet (2019) 51:237–44. doi: 10.1038/s41588-018-0307-5 PMC6358542 30643251

[21] Wootton RE Richmond RC Stuijfzand BG Lawn RB Sallis HM Taylor GMJ. Evidence for causal effects of lifetime smoking on risk for depression and schizophrenia: a Mendelian randomisation study. Psychol Med (2020) 50:2435–43. doi: 10.1017/S0033291719002678 PMC7610182 31689377

[22] Saunders GRB Wang X Chen F Jang S-K Liu M Wang C. Genetic diversity fuels gene discovery for tobacco and alcohol use. Nature (2022) 612:720–4. doi: 10.1038/s41586-022-05477-4 PMC9771818 36477530

[23] Yengo L Sidorenko J Kemper KE Zheng Z Wood AR Weedon MN. Meta-analysis of genome-wide association studies for height and body mass index in ∼700000 individuals of European ancestry. Hum Mol Genet (2018) 27:3641–9. doi: 10.1093/hmg/ddy271 PMC6488973 30124842

[24] van de Vegte YJ Said MA Rienstra M van der Harst P Verweij N. Genome-wide association studies and Mendelian randomization analyses for leisure sedentary behaviours. Nat Commun (2020) 11:1770. doi: 10.1038/s41467-020-15553-w 32317632 PMC7174427

[25] Balagué F Mannion AF Pellisé F Cedraschi C. Non-specific low back pain. Lancet Lond Engl (2012) 379:482–91. doi: 10.1016/S0140-6736(11)60610-7 21982256

[26] Vlaeyen JWS Maher CG Wiech K Van Zundert J Meloto CB Diatchenko L. Low back pain. Nat Rev Dis Primer (2018) 4:52. doi: 10.1038/s41572-018-0052-1 30546064

[27] Patrick N Emanski E Knaub MA. Acute and chronic low back pain. Med Clin North Am (2014) 98:777–89. doi: 10.1016/j.mcna.2014.03.005 24994051

[28] Jin P Xing Y Xiao B Wei Y Yan K Zhao J. Diabetes and intervertebral disc degeneration: A Mendelian randomization study. Front Endocrinol (2023) 14:1100874. doi: 10.3389/fendo.2023.1100874 PMC10011653 36926034

[29] Liu H Wang L Chen C Dong Z Yu S. Association between dietary niacin intake and migraine among American adults: national health and nutrition examination survey. Nutrients (2022) 14:3052. doi: 10.3390/nu14153052 35893904 PMC9330821

[30] Nievergelt CM Maihofer AX Klengel T Atkinson EG Chen C-Y Choi KW. International meta-analysis of PTSD genome-wide association studies identifies sex- and ancestry-specific genetic risk loci. Nat Commun (2019) 10:4558. doi: 10.1038/s41467-019-12576-w 31594949 PMC6783435

[31] McCartney DL Min JL Richmond RC Lu AT Sobczyk MK Davies G. Genome-wide association studies identify 137 genetic loci for DNA methylation biomarkers of aging. Genome Biol (2021) 22:194. doi: 10.1186/s13059-021-02398-9 34187551 PMC8243879

[32] Xu Z Zhang F Qiu G Shi Y Yu D Dai G. The causality of physical activity status and intelligence: A bidirectional Mendelian randomization study. PloS One (2023) 18:e0289252. doi: 10.1371/journal.pone.0289252 37527259 PMC10393173

[33] Bowden J Davey Smith G Burgess S. Mendelian randomization with invalid instruments: effect estimation and bias detection through Egger regression. Int J Epidemiol (2015) 44:512–25. doi: 10.1093/ije/dyv080 PMC4469799 26050253

[34] Skrivankova VW Richmond RC Woolf BAR Yarmolinsky J Davies NM Swanson SA. Strengthening the reporting of observational studies in epidemiology using mendelian randomization: the STROBE-MR statement. JAMA (2021) 326:1614–21. doi: 10.1001/jama.2021.18236 34698778

[35] Burgess S Butterworth A Thompson SG. Mendelian randomization analysis with multiple genetic variants using summarized data. Genet Epidemiol (2013) 37:658–65. doi: 10.1002/gepi.21758 PMC4377079 24114802

[36] Natarajan S Lipsitz SR Fitzmaurice GM Sinha D Ibrahim JG Haas J. An extension of the Wilcoxon Rank-Sum test for complex sample survey data. J R Stat Soc Ser C Appl Stat (2012) 61:653–64. doi: 10.1111/j.1467-9876.2011.01028.x PMC3729471 23913985

[37] Rao JNK Scott AJ. The analysis of categorical data from complex sample surveys: chi-squared tests for goodness of fit and independence in two-way tables. J Am Stat Assoc (1981) 76:221–30. doi: 10.1080/01621459.1981.10477633

[38] Sanderson E. Multivariable mendelian randomization and mediation. Cold Spring Harb Perspect Med (2021) 11:a038984. doi: 10.1101/cshperspect.a038984 32341063 PMC7849347

[39] Zhao SS Holmes MV Zheng J Sanderson E Carter AR. The impact of education inequality on rheumatoid arthritis risk is mediated by smoking and body mass index: Mendelian randomization study. Rheumatol Oxf Engl (2022) 61:2167–75. doi: 10.1093/rheumatology/keab654 PMC9071527 34436562

[40] Herrmann M Probst P Hornung R Jurinovic V Boulesteix A-L. Large-scale benchmark study of survival prediction methods using multi-omics data. Brief Bioinform (2021) 22:bbaa167. doi: 10.1093/bib/bbaa167 32823283 PMC8138887

[41] Burgess S Thompson SG. Interpreting findings from Mendelian randomization using the MR-Egger method. Eur J Epidemiol (2017) 32:377–89. doi: 10.1007/s10654-017-0255-x PMC5506233 28527048

[42] Verbanck M Chen C-Y Neale B Do R. Detection of widespread horizontal pleiotropy in causal relationships inferred from Mendelian randomization between complex traits and diseases. Nat Genet (2018) 50:693–8. doi: 10.1038/s41588-018-0099-7 PMC6083837 29686387

[43] Burgess S Davies NM Thompson SG. Bias due to participant overlap in two-sample Mendelian randomization. Genet Epidemiol (2016) 40:597–608. doi: 10.1002/gepi.21998 27625185 PMC5082560

[44] Harris LT Koepsell TD Haneuse SJ Martin DP Ralston JD. Glycemic control associated with secure patient-provider messaging within a shared electronic medical record: a longitudinal analysis. Diabetes Care (2013) 36:2726–33. doi: 10.2337/dc12-2003 PMC3747898 23628618

[45] Sterne JAC White IR Carlin JB Spratt M Royston P Kenward MG. Multiple imputation for missing data in epidemiological and clinical research: potential and pitfalls. BMJ (2009) 338:b2393. doi: 10.1136/bmj.b2393 19564179 PMC2714692

[46] Shmagel A Foley R Ibrahim H. Epidemiology of chronic low back pain in US adults: data from the 2009-2010 national health and nutrition examination survey. Arthritis Care Res (2016) 68:1688–94. doi: 10.1002/acr.22890 PMC5027174 26991822

[47] Ferreira PH Pinheiro MB MaChado GC Ferreira ML. Is alcohol intake associated with low back pain? A systematic review of observational studies. Man Ther (2013) 18:183–90. doi: 10.1016/j.math.2012.10.007 23146385

[48] Dionne C Koepsell TD Von Korff M Deyo RA Barlow WI Checkoway H. Formal education and back-related disability. In search of an explanation. Spine (1995) 20:2721–30. doi: 10.1097/00007632-199512150-00014 8747251

[49] Dionne CE Von Korff M Koepsell TD Deyo RA Barlow WE Checkoway H. Formal education and back pain: a review. J Epidemiol Community Health (2001) 55:455–68. doi: 10.1136/jech.55.7.455 PMC1731944 11413174

[50] Seiglie JA Marcus M-E Ebert C Prodromidis N Geldsetzer P Theilmann M. Diabetes prevalence and its relationship with education, wealth, and BMI in 29 low- and middle-income countries. Diabetes Care (2020) 43:767–75. doi: 10.2337/dc19-1782 PMC7085810 32051243

[51] Zhou J Mi J Peng Y Han H Liu Z. Causal associations of obesity with the intervertebral degeneration, low back pain, and sciatica: a two-sample mendelian randomization study. Front Endocrinol (2021) 12:740200. doi: 10.3389/fendo.2021.740200 PMC8692291 34956075

[52] Hess AS Hess JR. Logistic regression. Transfusion (2019) 59:2197–8. doi: 10.1111/trf.15406 31211435

[53] Wang L Ren J Chen J Gao R Bai B An H. Lifestyle choices mediate the association between educational attainment and BMI in older adults in China: A cross-sectional study. Front Public Health (2022) 10:1000953. doi: 10.3389/fpubh.2022.1000953 36388355 PMC9643852

[54] Assari S Bazargan M. Unequal associations between educational attainment and occupational stress across racial and ethnic groups. Int J Environ Res Public Health (2019) 16:3539. doi: 10.3390/ijerph16193539 31546681 PMC6801852

[55] Song Z Li W-D Li H Zhang X Wang N Fan Q. Genetic basis of job attainment characteristics and the genetic sharing with other SES indices and well-being. Sci Rep (2022) 12:8902. doi: 10.1038/s41598-022-12905-y 35618877 PMC9135765

[56] Hartvigsen J Hancock MJ Kongsted A Louw Q Ferreira ML Genevay S. What low back pain is and why we need to pay attention. Lancet Lond Engl (2018) 391:2356–67. doi: 10.1016/S0140-6736(18)30480-X 29573870

[57] Akmal M Kesani A Anand B Singh A Wiseman M Goodship A. Effect of nicotine on spinal disc cells: a cellular mechanism for disc degeneration. Spine (2004) 29:568–75. doi: 10.1097/01.brs.0000101422.36419.d8 15129075

[58] Shi Y Weingarten TN Mantilla CB Hooten WM Warner DO. Smoking and pain: pathophysiology and clinical implications. Anesthesiology (2010) 113:977–92. doi: 10.1097/ALN.0b013e3181ebdaf9 20864835

[59] Urquhart DM Berry P Wluka AE Strauss BJ Wang Y Proietto J. 2011 Young Investigator Award winner: Increased fat mass is associated with high levels of low back pain intensity and disability. Spine (2011) 36:1320–5. doi: 10.1097/BRS.0b013e3181f9fb66 21270692

[60] Boutens L Hooiveld GJ Dhingra S Cramer RA Netea MG Stienstra R. Unique metabolic activation of adipose tissue macrophages in obesity promotes inflammatory responses. Diabetologia (2018) 61:942–53. doi: 10.1007/s00125-017-4526-6 PMC6448980 29333574

[61] González-Cubero E González-Fernández ML Olivera ER Villar-Suárez V. Extracellular vesicle and soluble fractions of adipose tissue-derived mesenchymal stem cells secretome induce inflammatory cytokines modulation in an *in vitro* model of discogenic pain. Spine J Off J North Am Spine Soc (2022) 22:1222–34. doi: 10.1016/j.spinee.2022.01.012 35121152

