# Supplementary material for: Smoking and BMI mediate the causal effect of education on lower back pain: observational and Mendelian randomization analyses
Source: Front Endocrinol (Lausanne). 2024 Feb 6;15:1288170. doi: 10.3389/fendo.2024.1288170 (PMC10882710; doi:10.3389/fendo.2024.1288170)
Supplement: Supplementary file 1 [file DataSheet_1.docx]

Supplementary Material

**Smoking and BMI Mediate the Causal Effect of Education on Lower Back Pain: Observational and Mendelian Randomization Analyses**

Zhangmeng Xu^1,2^, Huiwu Zhang^2^, Duoduo Yu^2^, Luming Qi^1^, Yushan Shi^3^, Yaming Yu^2*^, Tianmin Zhu^1*^

Contents

[Supplementary STROBE-MR Checklist. 1](#_Toc152451797)

[Supplementary STROBE-cross-sectional studies Checklist. 5](#_Toc152451798)

[Supplementary Table S1. Summarized information for GWASs included 6](#_Toc152451799)

[Supplementary Table S2. Baseline data comparison of included and excluded participates 7](#_Toc152451800)

[Supplementary Table S3. Weighted association of covariates and low back pain risk 9](#_Toc152451801)

[Supplementary Table S4. Exploration of the weighted association among education, smoking, BMI, and LBP. 10](#_Toc152451802)

[Supplementary Table S5. Heterogeneity tests for UVMR analysis 11](#_Toc152451803)

[Supplementary Table S6. Horizontal pleiotropy test for UVMR analysis 12](#_Toc152451804)

[Supplementary Table S7. Result of other datasets validation 13](#_Toc152451805)

[Supplementary Table S8. Probability of bias and type I error in MR analysis 14](#_Toc152451806)

[Supplementary Table S9. Multiple interpolation datasets analysis 15](#_Toc152451807)

[Supplementary Table S10. Removing extreme values of BMI datasets analysis 16](#_Toc152451808)

[Supplementary Figure S1. Model-1 sensitivity test in multivariate MR analysis 17](#_Toc152451809)

[Supplementary Figure S2. Model-2 sensitivity test in multivariate MR analysis 18](#_Toc152451810)

[Supplementary Figure S3. Model-3 sensitivity test in multivariate MR analysis 19](#_Toc152451811)

[Supplementary Figure S4.Model-4 sensitivity test in multivariate MR analysis 20](#_Toc152451812)

[Supplementary Figure S5. Scatter plot of education on potential mediators. 21](#_Toc152451813)

[Supplementary Figure S6. Funnel plot of education on potential mediators. 22](#_Toc152451814)

[Supplementary Figure S7. Leave-one-out plot of education on potential mediators. 23](#_Toc152451815)

[Supplementary Figure S8. Forest map of education on potential mediators. 23](#_Toc152451816)

[Supplementary Figure S9. Sensitivity analysis of education to LBP. 24](#_Toc152451817)

# Supplementary STROBE-MR Checklist.

**Strengthening the Reporting of Observational Studies in Epidemiology using Mendelian Randomization (STROBE-MR) checklist**

| Item | Complete/location |
| --- | --- |
| 1. Title and Abstract  Indicate Mendelian randomization (MR) as the study’s design in the title and/or the abstract if that is a main purpose of the study | Title and abstract |
| Introduction |  |
| 2.Background  Explain the scientific background and rationale for the reported study. What is the exposure? Is a potential causal relationship between exposure and outcome plausible? Justify why MR is a helpful method to address the study question | Introduction, section of Paragraphs 1–3 |
| 3.Objectives  State specific objectives clearly, including pre-specified causal hypotheses (if any). State that MR is a method that, under specific assumptions, intends to estimate causal effects | Introduction, section of Paragraphs 4 |
| 4. Study design and data sources  Present key elements of the study design early in the article. Consider including a table listing sources of data for all phases of the study. For each data source contributing to the analysis, describe the following: | Materials and methods，section of Study design and data sources |
| a) Setting: Describe the study design and the underlying population, if possible. Describe the setting, locations, and relevant dates, including periods of recruitment, exposure, follow-up, and data collection, when available. | Materials and methods，section of Study design |
| b) Participants: Give the eligibility criteria, and the sources and methods of section of participants. Report the sample size, and whether any power or sample size calculations were carried out prior to the main analysis | Materials and methods，section of Data sources for Mendelian randomization |
| c) Describe measurement, quality control and selection of genetic variants | Materials and methods，section of instrumental variables |
| d) For each exposure, outcome, and other relevant variables, describe methods of assessment and diagnostic criteria for diseases | Materials and methods，section of Data sources for Mendelian randomization |
| e) Provide details of ethics committee approval and participant informed consent, if relevant | section of Ethics approval |
| 5. Assumptions  Explicitly state the three core IV assumptions for the main analysis (relevance, independence and exclusion restriction) as well assumptions for any additional or sensitivity analysis | Materials and methods，Selection of instrumental variables |
| 6. Statistical methods main analysis Describe statistical methods and statistics use | Materials and methods，section of Statistical analysis |
| a) Describe how quantitative variables were handled in the analyses (i.e., scale, units, model) | Materials and methods，section of Data sources for Mendelian randomization |
| b) Describe how genetic variants were handled in the analyses and, if applicable, how their weights were selected | Materials and methods，section of Selection of instrumental variables |
| c) Describe the MR estimator (e.g. two-stage least squares, Wald ratio) and related statistics. Detail the included covariates and, in case of two-sample MR, whether the same covariate set was used for adjustment in the two samples | Materials and methods，section of Statistical analysis, Paragraphs 1 |
| d) Explain how missing data were addressed | Materials and methods，section of Statistical analysis, Paragraphs 1 |
| e) If applicable, indicate how multiple testing was addressed | Materials and methods，last paragraph |
| 7. Assessment of assumptions  Describe any methods or prior knowledge used to assess the assumptions or justify their validity | Materials and methods，section of Statistical analysis |
| 8.Sensitivity analyses  Describe any sensitivity analyses or additional analyses performed (e.g. comparison of effect estimates from different approaches, independent replication, bias analytic techniques, validation of instruments, simulations) | Materials and methods，section of Sensitivity analysis |
| 9. Software and pre-registration a) Name statistical software and package(s), including version and settings used | Materials and methods，last paragraph |
| b) State whether the study protocol and details were pre-registered (as well as when and where) | Not applicable |
| Results |  |
| 10. Descriptive data a) Report the numbers of individuals at each stage of included studies and reasons for exclusion. Consider use of a flow diagram | Results, section of Instrumental variables and demographic characteristics, paragraph 1 |
| b) Report summary statistics for phenotypic exposure(s), outcome(s), and other relevant variables (e.g. means, SDs, proportions) | Not applicable |
| c) If the data sources include meta-analyses of previous studies, provide the assessments of heterogeneity across these studies | Not applicable |
| d) For two-sample MR:  i. Provide justification of the similarity of the genetic variant-exposure associations between the exposure and outcome samples ii. Provide information on the number of individuals who overlap between the exposure and outcome studies | Materials and methods，section of Data sources for Mendelian randomization |
| 11. Main results a) Report the associations between genetic variant and exposure, and between genetic variant and outcome, preferably on an interpretable scale | Results, section of Instrumental variables and demographic characteristics, paragraph 1 |
| b) Report MR estimates of the relationship between exposure and outcome, and the measures of uncertainty from the MR analysis, on an interpretable scale, such as odds ratio or relative risk per SD difference | Results, Fig.2, Fig 3 |
| c) If relevant, consider translating estimates of relative risk into absolute risk for a meaningful time period | Not applicable |
| d) Consider plots to visualize results (e.g. forest plot, scatterplot of associations between genetic variants and outcome versus between genetic variants and exposure) | Results, Fig.2, Fig 3, Supplementary figure s5-9 |
| 12. Assessment of assumptions a) Report the assessment of the validity of the assumptions | Results, section of The effects of education and potential mediators on LBP, paragraph 1; section of Mediating effect, paragraph 1. |
| b) Report any additional statistics (e.g., assessments of heterogeneity across genetic variants, such as I2, Q statistic or E-value) | Results, section of Sensitivity analysis, paragraph 1-2. |
| 13. Sensitivity and additional analyses a) Report any sensitivity analyses to assess the robustness of the main results to violations of the assumptions | Results, section of Sensitivity analysis, paragraph 1-2. |
| b) Report results from other sensitivity analyses or additional analyses | Supplementary Table S6. |
| c) Report any assessment of direction of causal relationship (e.g., bidirectional MR) | Results, Fig.2, Fig 3, |
| d) When relevant, report and compare with estimates from non-MR analyses | Results, section of Mediating effect,paragraph 3. |
| e) Consider additional plots to visualize results (e.g., leave-one-out analyses) | Results, section of Sensitivity analysis, paragraph 1-2.  Supplementary figure s1-9 |
| Discussion |  |
| 14. Key results  Summarize key results with reference to study objectives | Discussion paragraph 1 |
| 15. Limitations Discuss limitations of the study, taking into account the validity of the IV assumptions, other sources of potential bias, and imprecision. Discuss both direction and magnitude of any potential bias and any efforts to address them | Discussion paragraph 6 |
| 16. Interpretations a) Meaning: Give a cautious overall interpretation of results in the context of their limitations and in comparison with other studies | Discussion paragraph 2–4 |
| b) Mechanism: Discuss underlying biological mechanisms that could drive a potential causal relationship between the investigated exposure and the outcome, and whether the gene-environment equivalence assumption is reasonable. Use causal language carefully, clarifying that IV estimates may provide causal effects only under certain assumptions | Discussion paragraph 5 |
| c) Clinical relevance: Discuss whether the results have clinical or public policy relevance, and to what extent they inform effect sizes of possible interventions | Discussion paragraph 6 |
| 17. Generalizability  Discuss the generalizability of the study results (a) to other populations, (b) across other exposure periods/timings, and (c) across other levels of exposure | Discussion paragraph 4–6 |
| 18.  Funding  Describe sources of funding and the role of funders in the present study and, if applicable, sources of funding for the databases and original study or studies on which the present study is based | Funding |
| 19. Data and data sharing  Provide the data used to perform all analyses or report where and how the data can be accessed, and reference these sources in the article. Provide the statistical code needed to reproduce the results in the article, or report whether the code is publicly accessible and if so, where | Data availability statement |
| 20. Conflicts of Interest  All authors should declare all potential conflicts of interest | Competing interest statement |

Skrivankova VW, Richmond RC, Woolf BAR, et al. Strengthening the reporting of observational studies in epidemiology using mendelian randomisation (STROBE-MR): explanation and elaboration. Bmj. 2021;375:n2233

# Supplementary STROBE-cross-sectional studies Checklist.

STROBE Statement—Checklist of items that should be included in reports of ***cross-sectional studies***

|  | Item No | Recommendation | Complete/location |
| --- | --- | --- | --- |
| Title and abstract | 1 | (a) Indicate the study’s design with a commonly used term in the title or the abstract | Title |
|  |  | (b) Provide in the abstract an informative and balanced summary of what was done and what was found | Abstract |
| Introduction | | |  |
| Background/rationale | 2 | Explain the scientific background and rationale for the investigation being reported | Introduction, section of Paragraphs 1–3 |
| Objectives | 3 | State specific objectives, including any prespecified hypotheses | Introduction, section of Paragraphs 4 |
| Methods | | |  |
| Study design | 4 | Present key elements of study design early in the paper | Materials and methods，section of Study design |
| Setting | 5 | Describe the setting, locations, and relevant dates, including periods of recruitment, exposure, follow-up, and data collection | Materials and methods，section of Data sources |
| Participants | 6 | (a) Give the eligibility criteria, and the sources and methods of selection of participants | Materials and methods，section of Data for observational study, Paragraphs 1 |
| Variables | 7 | Clearly define all outcomes, exposures, predictors, potential confounders, and effect modifiers. Give diagnostic criteria, if applicable | Materials and methods，section of Data for observational study, Paragraphs 2 |
| Data sources/ measurement | 8* | For each variable of interest, give sources of data and details of methods of assessment (measurement). Describe comparability of assessment methods if there is more than one group | Materials and methods，section of Data for observational study, Paragraphs 2 |
| Bias | 9 | Describe any efforts to address potential sources of bias | Materials and methods，section of Statistical analysis, Paragraphs 2 |
| Study size | 10 | Explain how the study size was arrived at | Materials and methods，section of Data for observational study, Paragraphs 1 |
| Quantitative variables | 11 | Explain how quantitative variables were handled in the analyses. If applicable, describe which groupings were chosen and why | Materials and methods，section of Statistical analysis, Paragraphs 2 |
| Statistical methods | 12 | (a) Describe all statistical methods, including those used to control for confounding | Materials and methods，section of Statistical analysis, Paragraphs 2 |
|  |  | (b) Describe any methods used to examine subgroups and interactions | Materials and methods，section of Statistical analysis, Paragraphs 2 |
|  |  | (c) Explain how missing data were addressed | Materials and methods，section of Statistical analysis, Paragraphs 2 |
|  |  | (d) If applicable, describe analytical methods taking account of sampling strategy | Not applicable |
|  |  | (e) Describe any sensitivity analyses | Materials and methods，section of Sensitivity analysis, Paragraphs 2 |
| Results | | |  |
| Participants | 13* | (a) Report numbers of individuals at each stage of study—eg numbers potentially eligible, examined for eligibility, confirmed eligible, included in the study, completing follow-up, and analysed | Results, section of Instrumental variables and demographic characteristics, paragraph 2, Fig 1 |
|  |  | (b) Give reasons for non-participation at each stage | Results, section of Instrumental variables and demographic characteristics, paragraph 2, Fig 1 |
|  |  | (c) Consider use of a flow diagram | Fig 1 |
| Descriptive data | 14* | (a) Give characteristics of study participants (eg demographic, clinical, social) and information on exposures and potential confounders | Table 1 |
|  |  | (b) Indicate number of participants with missing data for each variable of interest | Supplementary Table S1 |
| Outcome data | 15* | Report numbers of outcome events or summary measures | Results, section of Instrumental variables and demographic characteristics, paragraph 2, Fig 1 |
| Main results | 16 | (a) Give unadjusted estimates and, if applicable, confounder-adjusted estimates and their precision (eg, 95% confidence interval). Make clear which confounders were adjusted for and why they were included | Results, section of The effects of education and potential mediators on LBP, paragraph 2-3, Table 2 |
|  |  | (b) Report category boundaries when continuous variables were categorized | Materials and methods，section of Statistical analysis, Paragraphs 2 |
|  |  | (c) If relevant, consider translating estimates of relative risk into absolute risk for a meaningful time period | Not applicable |
| Other analyses | 17 | Report other analyses done—eg analyses of subgroups and interactions, and sensitivity analyses | Results, section of The effects of education and potential mediators on LBP, paragraph 4, Fig 3 |
| Discussion | | |  |
| Key results | 18 | Summarise key results with reference to study objectives | Results, section of Instrumental variables and demographic characteristics, paragraph 1 |
| Limitations | 19 | Discuss limitations of the study, taking into account sources of potential bias or imprecision. Discuss both direction and magnitude of any potential bias | Discussion paragraph 6 |
| Interpretation | 20 | Give a cautious overall interpretation of results considering objectives, limitations, multiplicity of analyses, results from similar studies, and other relevant evidence | Discussion paragraph 2–5 |
| Generalisability | 21 | Discuss the generalisability (external validity) of the study results | Discussion paragraph 6 |
| Other information | | |  |
| Funding | 22 | Give the source of funding and the role of the funders for the present study and, if applicable, for the original study on which the present article is based | Funding |

*Give information separately for cases and controls.

**Note:** An Explanation and Elaboration article discusses each checklist item and gives methodological background and published examples of transparent reporting. The STROBE checklist is best used in conjunction with this article (freely available on the Web sites of PLoS Medicine at http://www.plosmedicine.org/, Annals of Internal Medicine at http://www.annals.org/, and Epidemiology at http://www.epidem.com/). Information on the STROBE Initiative is available at www.strobe-statement.org.

# Supplementary Table S. Summarized information for GWASs included

| Study | Authors | PMID | Sample size | No. of SNPs | R2 (%) | F |
| --- | --- | --- | --- | --- | --- | --- |
| Educational | Lee et al. | 30038396 | 766,345 | 317 | 8 | 19.6 |
| Low back pain | Finngen | / | 177,860 |  |  |  |
| Smoking | Liu et al. | 30643251 | 1,200,000 | 123 | 10 | 17.76 |
| Alcohol consumption | Saunders et al. | 36477530 | 2,669,029 | 98 | 6 | 16.85 |
| BMI | Yengo et al. | 30124842 | 700,000 | 521 | 2.1 | 29.25 |
| leisure television | Van et al. | 32317632 | 422,218 | 148 | 6 | 17.05 |

GWAS, Genome-Wide Association Studies, BMI, body mass index.

Note: R^2^ = $2\times\left(1-EAF\right)\times EAF\times\frac{\text{β}^{2}}{SD^{2}}$; F = $\frac{N-k-1}{k}\times\frac{R^{2}}{1-R^{2}}$ (1,2)

# Supplementary Table S. Baseline data comparison of included and excluded participates

| Characteristic | Overall,  N^1^ = 200,707,729 | Exclude,  N^1^ = 51756209 | Include,  N^1^ = 148951520 | *P* Value^2^ |
| --- | --- | --- | --- | --- |
| Age (y), Mean ± SD | 46.2±17.0 | 46.8±18.7 | 46.0±16.4 | 0.9 |
| Gender, n (%) |  |  |  | <0.001 |
| Male | 95,989,894 (48%) | 22,139,666 (43%) | 73,850,228 (50%) |  |
| Female | 104,717,834 (52%) | 29,616,543 (57%) | 75,101,291 (50%) |  |
| BMI (kg/m2), Mean ± SD | 28.1±6.3 | 28.1±6.4 | 28.1±6.3 | 0.8 |
| Missing | 17,981,234 | 17,981,234 | 0 |  |
| Race, n (%) |  |  |  | <0.001 |
| Non-Hispanic white | 144,027,823 (72%) | 34,117,960 (66%) | 109,909,863 (74%) |  |
| Non-Hispanic black | 22,234,010 (11%) | 6,786,815 (13%) | 15,447,196 (10%) |  |
| Mexican American | 14,629,707 (7.3%) | 4,110,092 (7.9%) | 10,519,616 (7.1%) |  |
| Others | 19,816,188 (9.9%) | 6,741,343 (13%) | 13,074,845 (8.8%) |  |
| Education level, n (%) |  |  |  | <0.001 |
| Less than high school | 41,749,982 (21%) | 13,521,000 (26%) | 28,228,982 (19%) |  |
| High school diploma | 52,113,187 (26%) | 13,161,319 (26%) | 38,951,869 (26%) |  |
| More than high school | 106,275,691 (53%) | 24,505,023 (48%) | 81,770,669 (55%) |  |
| Missing | 568,868 | 568,868 | 0 |  |
| Marital status, n (%) |  |  |  | <0.001 |
| Married or living with a partner | 122,269,690 (64%) | 24,848,689 (57%) | 97,421,000 (65%) |  |
| Living alone | 70,210,573 (36%) | 18,680,054 (43%) | 51,530,519 (35%) |  |
| Missing | 8,227,466 | 8,227,466 | 0 |  |
| Family income, n (%) |  |  |  | 0.002 |
| Low | 39,832,533 (22%) | 8,750,179 (25%) | 31,082,354 (21%) |  |
| Medium | 66,754,198 (36%) | 13,354,547 (38%) | 53,399,652 (36%) |  |
| High | 77,209,187 (42%) | 12,739,672 (37%) | 64,469,515 (43%) |  |
| Missing | 16,911,811 | 16,911,811 | 0 |  |
| Alcohol consumption, n (%) |  |  |  | 0.001 |
| No | 49,064,410 (28%) | 8,580,300 (34%) | 40,484,110 (27%) |  |
| Yes | 125,190,428 (72%) | 16,723,019 (66%) | 108,467,410 (73%) |  |
| Missing | 26,452,891 | 26,452,891 | 0 |  |
| Activity, n (%) |  |  |  | <0.001 |
| Sedentary | 76,528,526 (38%) | 23,832,063 (46%) | 52,696,463 (35%) |  |
| Moderate | 57,443,009 (29%) | 13,243,780 (26%) | 44,199,228 (30%) |  |
| Vigorous | 66,710,957 (33%) | 14,655,129 (28%) | 52,055,828 (35%) |  |
| Missing | 25,237 | 25,237 | 0 |  |
| Smoke, n (%) |  |  |  | 0.2 |
| Never | 100,459,933 (50%) | 26,778,672 (52%) | 73,681,261 (49%) |  |
| Former | 50,095,789 (25%) | 12,446,960 (24%) | 37,648,829 (25%) |  |
| Current | 49,787,570 (25%) | 12,166,139 (24%) | 37,621,430 (25%) |  |
| Missing | 364,438 | 364,438 | 0 |  |
| Hypertension or Diabetes, n (%) |  |  |  | 0.3 |
| No | 149,167,517 (75%) | 38,265,463 (76%) | 110,902,054 (74%) |  |
| Yes | 50,287,806 (25%) | 12,238,341 (24%) | 38,049,465 (26%) |  |
| Missing | 1,252,405 | 1,252,405 | 0 |  |
| Watching TV time (hours per day),  Median (IQR) | 2 (1, 5) | 2 (1, 5) | 2 (1, 5) | 0.109 |
| Missing | 113,648 | 113,648 | 0 |  |
| Low back pain, n (%) |  |  |  | 0.14 |
| No | 122,950,777 (61%) | 32,236,825 (62%) | 90,713,952 (61%) |  |
| Yes | 77,673,623 (39%) | 19,436,055 (38%) | 58,237,568 (39%) |  |
| Missing | 83,329 | 83,329 | 0 |  |
| ^1^  Weighted number of participants | | | | |
| ^2^ Wilcoxon rank-sum test for complex survey samples; chi-squared test with Rao & Scott's second-order correction | | | | |
| BMI, body mass index; SD, standard deviation; IQR, interquartile range. | | | | |

# Supplementary Table S. Weighted association of covariates and low back pain risk

| Characteristic | OR1 | 95% CI1 | P Value |
| --- | --- | --- | --- |
| Age (y) | 1.00 | 1.00, 1.01 | 0.063 |
| Gender, n(%) |  |  | 0.003 |
| Male | 1 (reference) | 1 (reference) |  |
| Female | 1.14 | 1.05, 1.25 |  |
| BMI (kg/m2) | 1.03 | 1.02, 1.03 | <0.001 |
| Race, n(%) |  |  | <0.001 |
| Non-Hispanic white | 1 (reference) | 1 (reference) |  |
| Non-Hispanic black | 0.86 | 0.75, 0.98 |  |
| Mexican American | 0.74 | 0.65, 0.84 |  |
| Others | 0.88 | 0.70, 1.11 |  |
| Educational level |  |  | <0.001 |
| Less than high school | 1 (reference) | 1 (reference) |  |
| High school diploma | 0.93 | 0.80, 1.08 |  |
| More than high school | 0.63 | 0.57, 0.71 |  |
| Marital status, n (%) |  |  | 0.926 |
| Married or living with a partner | 1 (reference) | 1 (reference) |  |
| Living alone | 1.00 | 0.91, 1.09 |  |
| Family income, n (%) |  |  | <0.001 |
| Low | 1 (reference) | 1 (reference) |  |
| Medium | 0.77 | 0.68, 0.87 |  |
| High | 0.59 | 0.51, 0.69 |  |
| Alcohol consumption, n (%) |  |  | 0.313 |
| No | 1 (reference) | 1 (reference) |  |
| Yes | 0.95 | 0.86, 1.05 |  |
| Activity, n (%) |  |  | <0.001 |
| Sedentary | 1 (reference) | 1 (reference) |  |
| Moderate | 0.84 | 0.74, 0.95 |  |
| Vigorous | 0.69 | 0.63, 0.76 |  |
| Smoke, n (%) |  |  | <0.001 |
| Never | 1 (reference) | 1 (reference) |  |
| Former | 1.22 | 1.07, 1.39 |  |
| Current | 1.47 | 1.29, 1.68 |  |
| Hypertension or Diabetes, n (%) |  |  | <0.001 |
| No | 1 (reference) | 1 (reference) |  |
| Yes | 1.47 | 1.32, 1.64 |  |
| Watching TV time (hours per day) | 1.05 | 1.02, 1.08 | <0.001 |

Note: Univariate logistic regression results. BMI, body mass index; OR, odd ratio.

# Supplementary Table S. Exploration of the weighted association among education, smoking, BMI, and LBP.

| Observed variables | Response variables | Event (%) | OR (95 CI%) | p-value |
| --- | --- | --- | --- | --- |
| High school level or below | LBP | 2353 (40.4) | 1(Ref) |  |
| Above high school level* | LBP | 1649 (34.7) | 0.74 (0.67~0.81) | <0.001 |
| Above high school level& | LBP& | 1649 (34.7) | 0.75 (0.69~0.83) | <0.001 |
| High school level or below | Smoke status | 3149 (54.1) | 1(Ref) |  |
| Above high school level | Smoke status | 2140 (45.0) | 0.62 (0.56~0.68) | <0.001 |
| High school level or below | BMI | 5822 (55.0) | 0(Ref) |  |
| Above high school level | BMI | 4758 (45.0） | β= -0.16 (-0.47~0.15) | 0.308 |
| Never smoked | LBP | 1839 (34.8) | 1(Ref) |  |
| Former or current smoker | LBP | 2163 (40.9) | 1.24 (1.10~1.40) | <0.001 |
| BMI | LBP | 4002 (37.8) | 1.02 (1.01~1.03) | <0.001 |

*: Unadjusted smoke status and BMI in model；&: Adjusted smoke status and BMI in model; Observed variables without superscripts adjusted for all covariates except their own. LBP, low back pain; BMI, body mass index; OR, odd ratio.

Note: The educational level was reclassified into two categories: high school level or below, and above high school level. Similarly, smoking status was categorized as never smoked, former or current smoker. When BMI is treated as a response variable, multivariate linear regression is used to determine the effect size, which is denoted by β.

# Supplementary Table S. Heterogeneity tests for UVMR analysis

| Exposure | Outcome | Methods | Q | Q-df | Q-pval |
| --- | --- | --- | --- | --- | --- |
| Education | LBP | MR Egger | 444.22 | 303 | 2.15E-07 |
| Education | LBP | IVW | 444.22 | 304 | 2.61E-07 |
| Education | Smoking Index | MR Egger | 1015.37 | 304 | 1.89E-77 |
| Education | Smoking Index | IVW | 1015.38 | 305 | 3.45E-77 |
| Education | Alcohol Consumption | MR Egger | 1073.48 | 303 | 1.05E-86 |
| Education | Alcohol Consumption | IVW | 1074.13 | 304 | 1.57E-86 |
| Education | BMI | MR Egger | 1168.41 | 140 | 9.95E-162 |
| Education | BMI | IVW | 1168.83 | 141 | 2.40E-161 |
| Education | Leisure Television | MR Egger | 840.89 | 305 | 1.15E-51 |
| Education | Leisure Television | IVW | 841.99 | 306 | 1.35E-51 |
| Smoking Index | LBP | MR Egger | 161.32 | 113 | 1.94E-03 |
| Smoking Index | LBP | IVW | 162.00 | 114 | 2.11E-03 |
| Alcohol Consumption | LBP | MR Egger | 143.58 | 91 | 3.67E-04 |
| Alcohol Consumption | LBP | IVW | 144.31 | 92 | 4.07E-04 |
| BMI | LBP | MR Egger | 658.64 | 492 | 6.85E-07 |
| BMI | LBP | IVW | 659.16 | 493 | 7.44E-07 |
| Leisure Television | LBP | MR Egger | 143.62 | 134 | 2.69E-01 |
| Leisure Television | LBP | IVW | 143.82 | 135 | 2.86E-01 |

BMI, body mass index; LBP, low back pain; IVW, inverse variance weighting.

Note: Q, Cochran's Q statistic in IVW and MR-Egger; df, (number of) degrees of freedom; Q-pval, the null hypothesis (H0) for Q-pval is that there is no difference between each SNP. UVMR, univariate Mendelian randomization.

# Supplementary Table S. Horizontal pleiotropy test for UVMR analysis

| Exposure | Outcome | Egger-intercept | SE | *p*-value | Distortion *p*-value |
| --- | --- | --- | --- | --- | --- |
| Education | LBP | 0.000 | 0.004 | 0.959 | 0.709 |
| Education | Smoking Index | 0.000 | 0.001 | 0.968 | 0.579 |
| Education | Alcohol Consumption | 0.000 | 0.001 | 0.668 | 0.026* |
| Education | BMI | 0.000 | 0.002 | 0.823 | 0.609 |
| Education | Leisure Television | 0.001 | 0.001 | 0.528 | 0.869 |
| Smoking Index | LBP | 0.004 | 0.006 | 0.490 | NA |
| Alcohol Consumption | LBP | -0.003 | 0.005 | 0.498 | 0.139 |
| BMI | LBP | 0.001 | 0.002 | 0.533 | 0.752 |
| Leisure Television | LBP | -0.002 | 0.005 | 0.665 | 0.165 |
| *no significant change in estimate after outlier removal; UVMR, univariate Mendelian randomization; BMI, body mass index; LBP, low back pain; SE, standard error. | | | | | |

Note: Egger-intercept represents the intercept computed using the MR-Egger method. The p-value associated with Egger-intercept refers to the hypothesis test conducted on the intercept, where the null hypothesis is that the intercept is equal to zero. Distortion *p*-value in MR-PRESSO represents the *p*-value of the distortion test. The null hypothesis for the distortion test is that there is no distortion in the MR estimation results caused by SNP-level horizontal pleiotropy.

# Supplementary Table S. Result of other datasets validation

| Models | exposure | outcome | nsnp | OR | OR-lci95 | OR-uci95 | *p*-value |
| --- | --- | --- | --- | --- | --- | --- | --- |
| Model-1 | Education | LBP | 26 | 0.992 | 0.985 | 0.999 | 0.027 |
|  | Smoking status | LBP | 5 | 1.027 | 1.001 | 1.054 | 0.038 |
|  | BMI | LBP | 388 | 1.003 | 1.001 | 1.005 | 0.012 |
|  | TV time | LBP | 37 | 1.012 | 1.003 | 1.022 | 0.013 |
| Model-2 | Education | LBP | 26 | 0.988 | 0.982 | 0.993 | <0.001 |
|  | Smoking status | LBP | 4 | 1.028 | 1.002 | 1.055 | 0.037 |
|  | BMI | LBP | 398 | 1.004 | 1.002 | 1.007 | <0.001 |
| Model-3 | Education | LBP | 67 | 0.990 | 0.986 | 0.995 | <0.001 |
|  | Smoking status | LBP | 12 | 1.046 | 1.014 | 1.080 | 0.004 |
| Model-4 | Education | LBP | 26 | 0.986 | 0.981 | 0.991 | <0.001 |
|  | BMI | LBP | 400 | 1.005 | 1.003 | 1.007 | <0.001 |

BMI, body mass index; LBP, low back pain; OR, odd ratio.

Notes: All data are sourced from IEU Open GWAS (https://gwas.mrcieu.ac.uk/). Educational attainment (ieu-a-1001) is derived from the study conducted by Okbay A et al (3), encompassing a sample size of 293,723 individuals, with each standard deviation representing an equivalent of 3.71 years of schooling. Smoking status (ukb-a-225) is obtained from the data released by Neale Labs in 2017 and represents the current smoking behavior as a dichotomous variable, comprising of 33,928 cases and 302,096 controls. BMI (ukb-b-2303) is presented as a continuous variable published by MRC-IEU in 2018 with a sample size of 454,884 individuals. TV time (ukb-b-5192) was also published by MRC-IEU in 2018 as an continuous variable (Time spent watching television) with a sample size of 437887. The LBP (ukb-d-M13-LOWBACKPAIN, https://www.finngen.fi/fi) data were obtained from a Finnish database containing 5423 cases and 355,771 controls. The diagnosis of the case was based on the WHO's International Classification of Diseases (ICD) inclusion criteria (ICD-10M54.5, ICD-9 7242, ICD-8 72870). All samples are of European ancestry.

# Supplementary Table S8. Probability of bias and type I error in MR analysis

| **Exposure** | **Outcome** | **Overlap cohorts** | **Overlap samples** | **Overlap proportion** | **Bias** | **Type 1 error rate** |
| --- | --- | --- | --- | --- | --- | --- |
| Education | Smoking | 23andMe | 365538 | 32.3% | 0.002 | 0.06 |
| Education | Alcohol consumption | EGCUT, UKB, WLS | 486603 | 43.0% | 0.002 | 0.08 |
| Education | BMI | EGCUT | 2610 | 0.3% | 0 | 0.05 |
| Education | Leisure Television | UKB | 442183 | 39.1% | 0.002 | 0.05 |
| Education | LBP | FENLAND | 8535 | 4.8% | 0 | 0.05 |
| Smoking | LBP | FinnTwin, NAG-FIN | 3058 | 1.72% | 0 | 0.05 |
| Alcohol consumption | LBP | FinnTwin, NAG-FIN, FINRISK | 26785 | 15.1% | 0 | 0.05 |
| BMI | LBP | FinGesture cases | 943 | 0.53% | 0 | 0.05 |
| Leisure Television | LBP | / | / | / | 0 | 0.05 |

BMI, body mass index; LBP, low back pain.

Note: The methods for assessing bias and Type I errors are derived from Burgess et al. (4).

# Supplementary Table S9. Multiple interpolation datasets analysis

| Education level |  | Crude | | Model-1 | | Model-2 | |
| --- | --- | --- | --- | --- | --- | --- | --- |
|  | NO.(case %) | OR (95% CI) | *p*-value | OR (95% CI) | *p*-value | OR (95% CI) | *p*-value |
| Less than high school | 21165 (39.6) | 1(Ref) |  | 1(Ref) |  | 1(Ref) |  |
| High school diploma | 15480 (40.5) | 1.04 (0.99~1.08) | 0.099 | 0.96 (0.91~1) | 0.052 | 0.97 (0.93~1.02) | 0.225 |
| More than high school | 27970 (34.7) | 0.81 (0.78~0.84) | <0.001 | 0.77 (0.74~0.81) | <0.001 | 0.81 (0.77~0.85) | <0.001 |
| Trend.test | 64615 (37.7) |  | <0.001 |  | <0.001 |  | <0.001 |

| Education level |  | Model-3 | | Model-4 | | Model-5 | |
| --- | --- | --- | --- | --- | --- | --- | --- |
|  | NO.(case %) | OR (95% CI) | *p*-value | OR (95% CI) | *p*-value | OR (95% CI) | *p*-value |
| Less than high school | 21165 (39.6) | 1(Ref) |  | 1(Ref) |  | 1(Ref) |  |
| High school diploma | 15480 (40.5) | 0.98 (0.93~1.02) | 0.289 | 0.96 (0.92~1.01) | 0.108 | 0.97 (0.92~1.01) | 0.148 |
| More than high school | 27970 (34.7) | 0.83 (0.8~0.87) | <0.001 | 0.81 (0.77~0.84) | <0.001 | 0.83 (0.8~0.87) | <0.001 |
| Trend test | 64615 (37.7) |  | <0.001 |  | <0.001 |  | <0.001 |

OR, odd ratio.

Note: Model-1 adjusts for age, gender, race, marital status, family income. Model-2 adjusts for model-1 + watching TV time, alcohol consumption, physical activity, and hypertension or diabetes status. Model-3 adjusts for model-2 + smoking status. Model-4 adjusts for model-2 + body mass index. Model-5 adjusts for model-2 + smoking status and body mass index.

# Supplementary Table S10. Removing extreme values of BMI datasets analysis

| Education level |  | Crude | | Model-1 | | Model-2 | |
| --- | --- | --- | --- | --- | --- | --- | --- |
|  | NO.(case %) | OR (95% CI) | *p*-value | OR (95% CI) | *p*-value | OR (95% CI) | *p*-value |
| Less than high school | 3088 (38.7) | 1(Ref) |  | 1(Ref) |  | 1(Ref) |  |
| High school diploma | 2356 (40.4) | 1.07 (0.96~1.2) | 0.21 | 0.97 (0.86~1.10) | 0.662 | 0.99 (0.87~1.11) | 0.817 |
| More than high school | 4446 (34.0) | 0.82 (0.74~0.90) | <0.001 | 0.77 (0.69~0.86) | <0.001 | 0.81 (0.72~0.91) | <0.001 |
| Trend.test | 9890 (37.0) |  | <0.001 |  | <0.001 |  | <0.001 |

| Education level |  | Model-3 | | Model-4 | | Model-5 | |
| --- | --- | --- | --- | --- | --- | --- | --- |
|  | NO.(case %) | OR (95% CI) | *p*-value | OR (95% CI) | *p*-value | OR (95% CI) | *p*-value |
| Less than high school | 3088(38.7) | 1(Ref) |  | 1(Ref) |  | 1(Ref) |  |
| High school diploma | 2356 (40.4) | 0.99 (0.88~1.12) | 0.894 | 0.98 (0.87~1.1) | 0.727 | 0.98 (0.87~1.11) | 0.801 |
| More than high school | 4446 (34.0) | 0.83 (0.74~0.93) | 0.002 | 0.81 (0.72~0.91) | <0.001 | 0.83 (0.74~0.93) | 0.002 |
| Trend.test | 9890 (37.0) |  | 0.001 |  | <0.001 |  | 0.001 |

OR, odd ratio.

Note: Model-1 adjusts for age, gender, race, marital status, family income. Model-2 adjusts for model-1 + watching TV time, alcohol consumption, physical activity, and hypertension or diabetes status. Model-3 adjusts for model-2 + smoking status. Model-4 adjusts for model-2 + body mass index. Model-5 adjusts for model-2 + smoking status and body mass index.


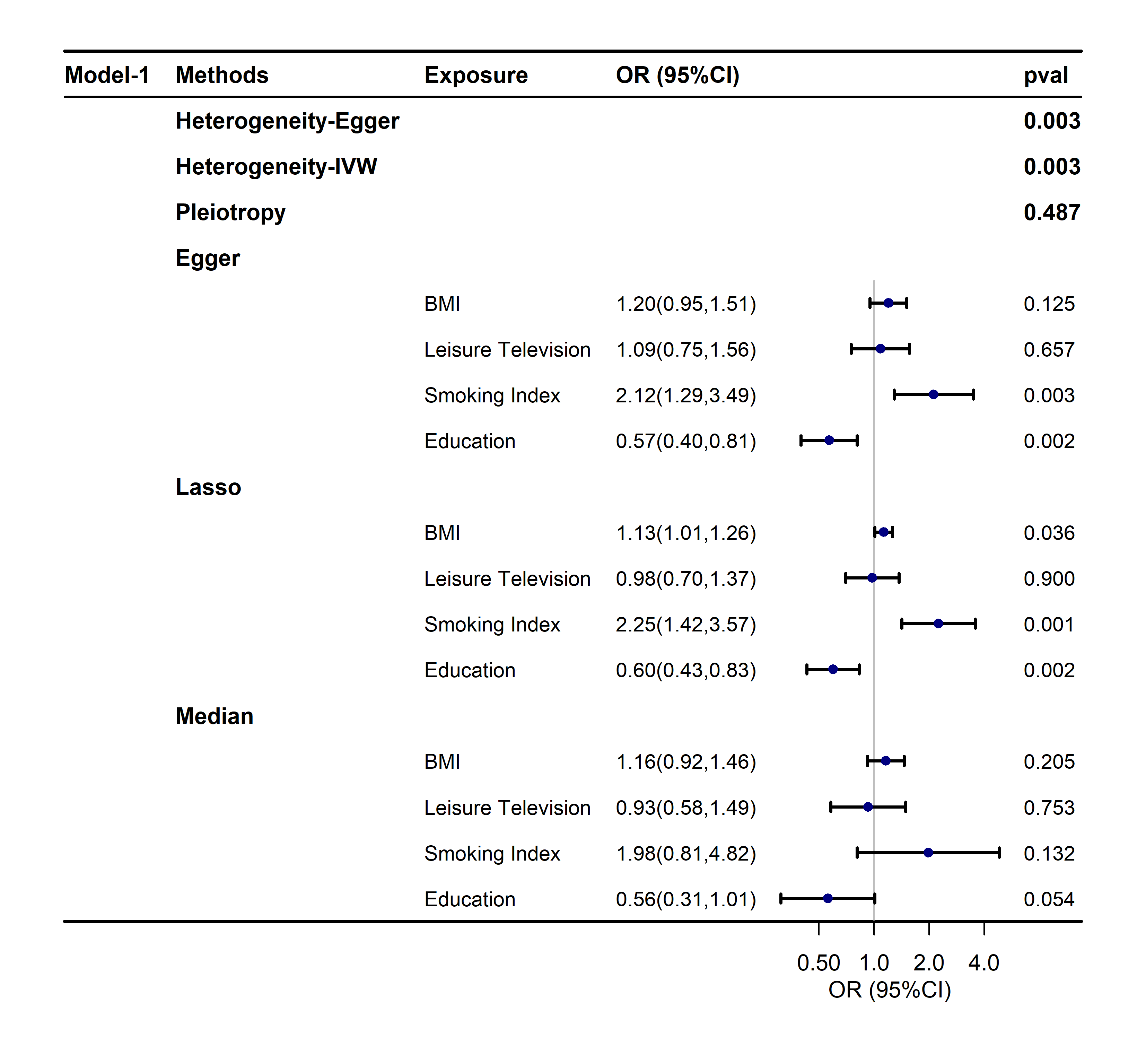


# Supplementary Figure S. Model-1 sensitivity test in multivariate MR analysis

Heterogeneity is obtained by hypothesis testing of Cochran's Q statistic in IVW and MR-Egger, and pleiotropy is obtained by hypothesis testing of intercept in MR-Egger. BMI, body mass index; OR, odd ratio.


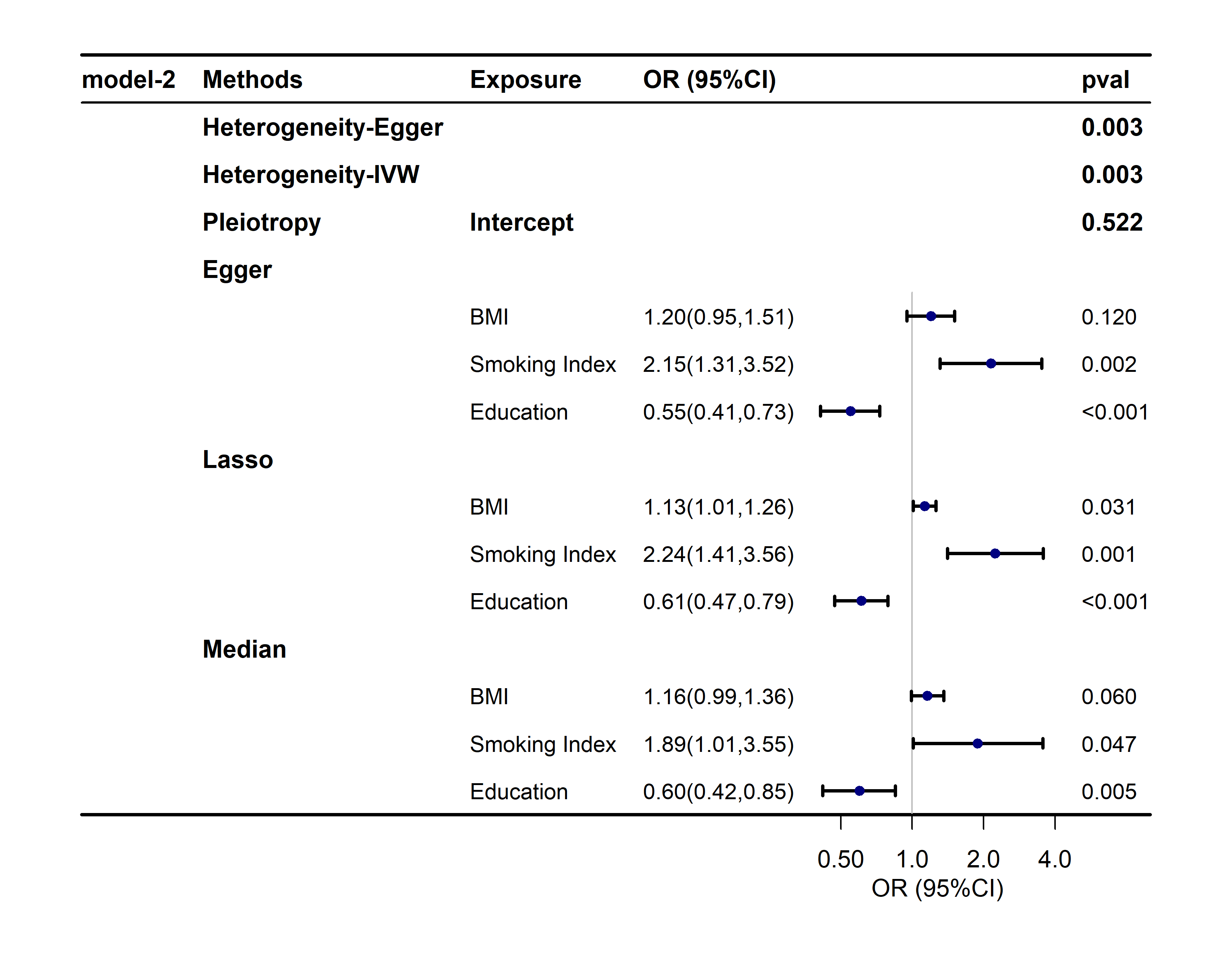


# Supplementary Figure S. Model-2 sensitivity test in multivariate MR analysis

Heterogeneity is obtained by hypothesis testing of Cochran's Q statistic in IVW and MR-Egger, and pleiotropy is obtained by hypothesis testing of intercept in MR-Egger. BMI, body mass index; OR, odd ratio.


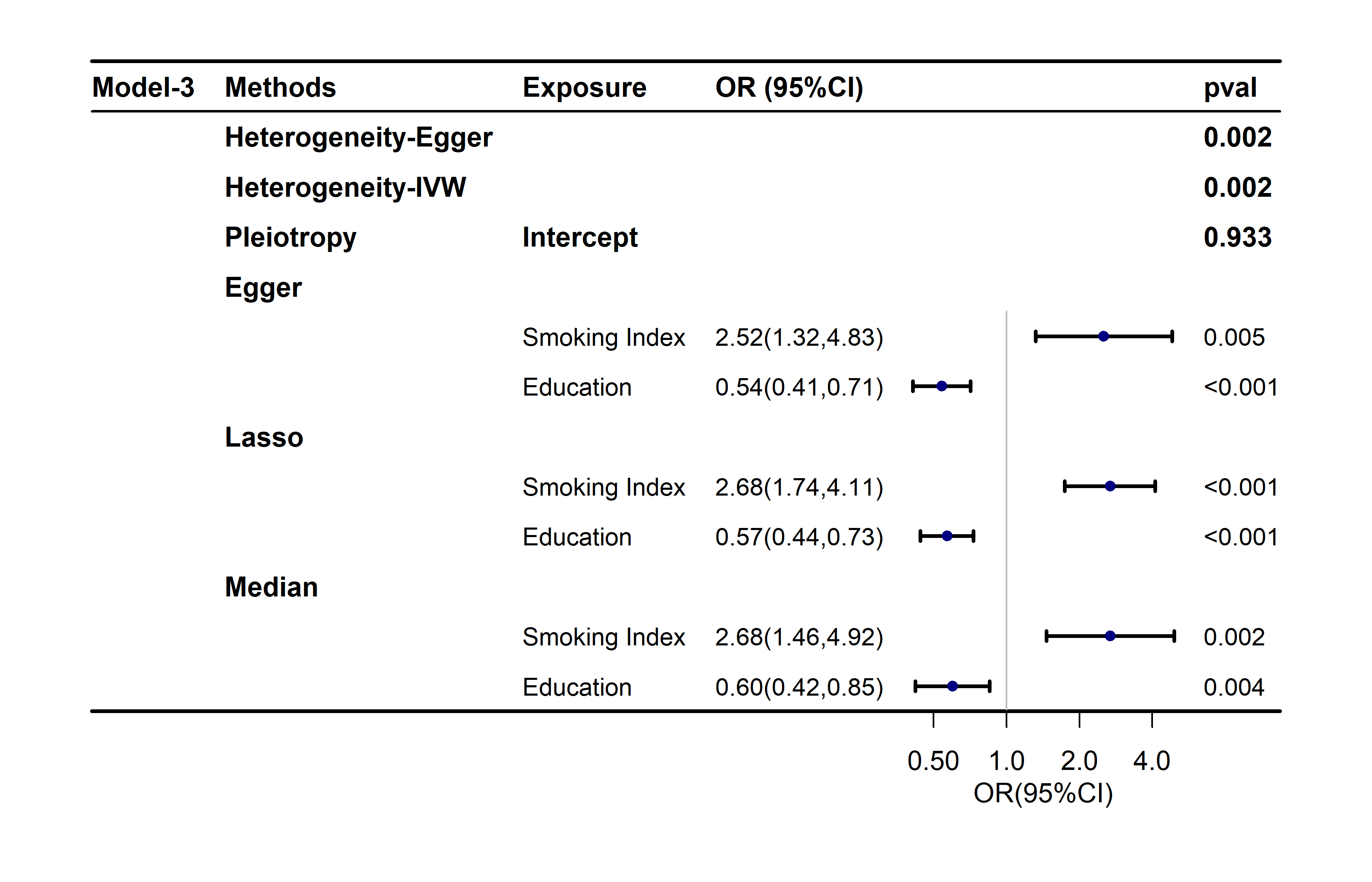


# Supplementary Figure S. Model-3 sensitivity test in multivariate MR analysis

Heterogeneity is obtained by hypothesis testing of Cochran's Q statistic in IVW and MR-Egger, and pleiotropy is obtained by hypothesis testing of intercept in MR-Egger. BMI, body mass index; OR, odd ratio.


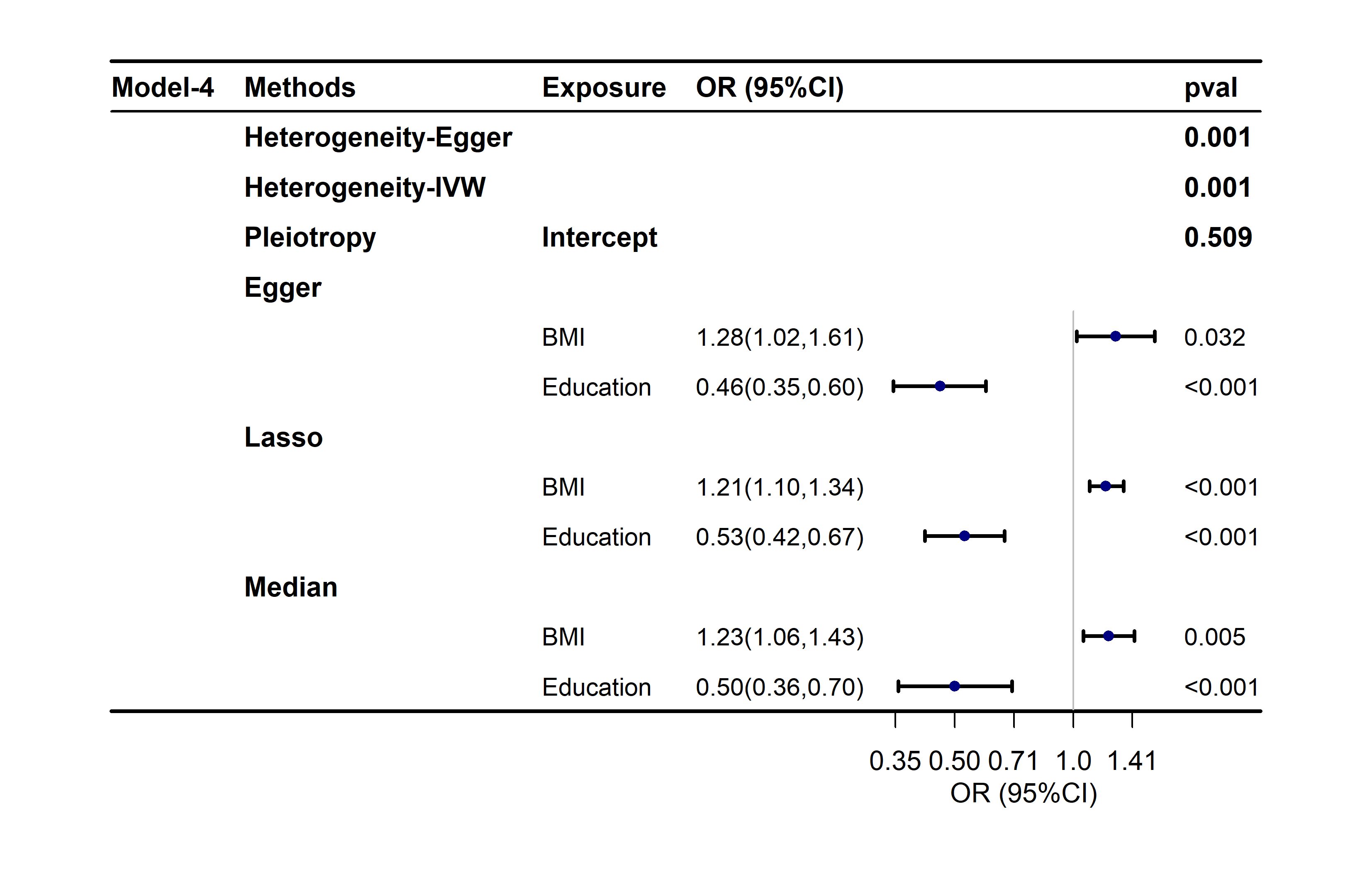


# Supplementary Figure S.Model-4 sensitivity test in multivariate MR analysis

Heterogeneity is obtained by hypothesis testing of Cochran's Q statistic in IVW and MR-Egger, and pleiotropy is obtained by hypothesis testing of intercept in MR-Egger. BMI, body mass index; OR, odd ratio.

**A**

**B**


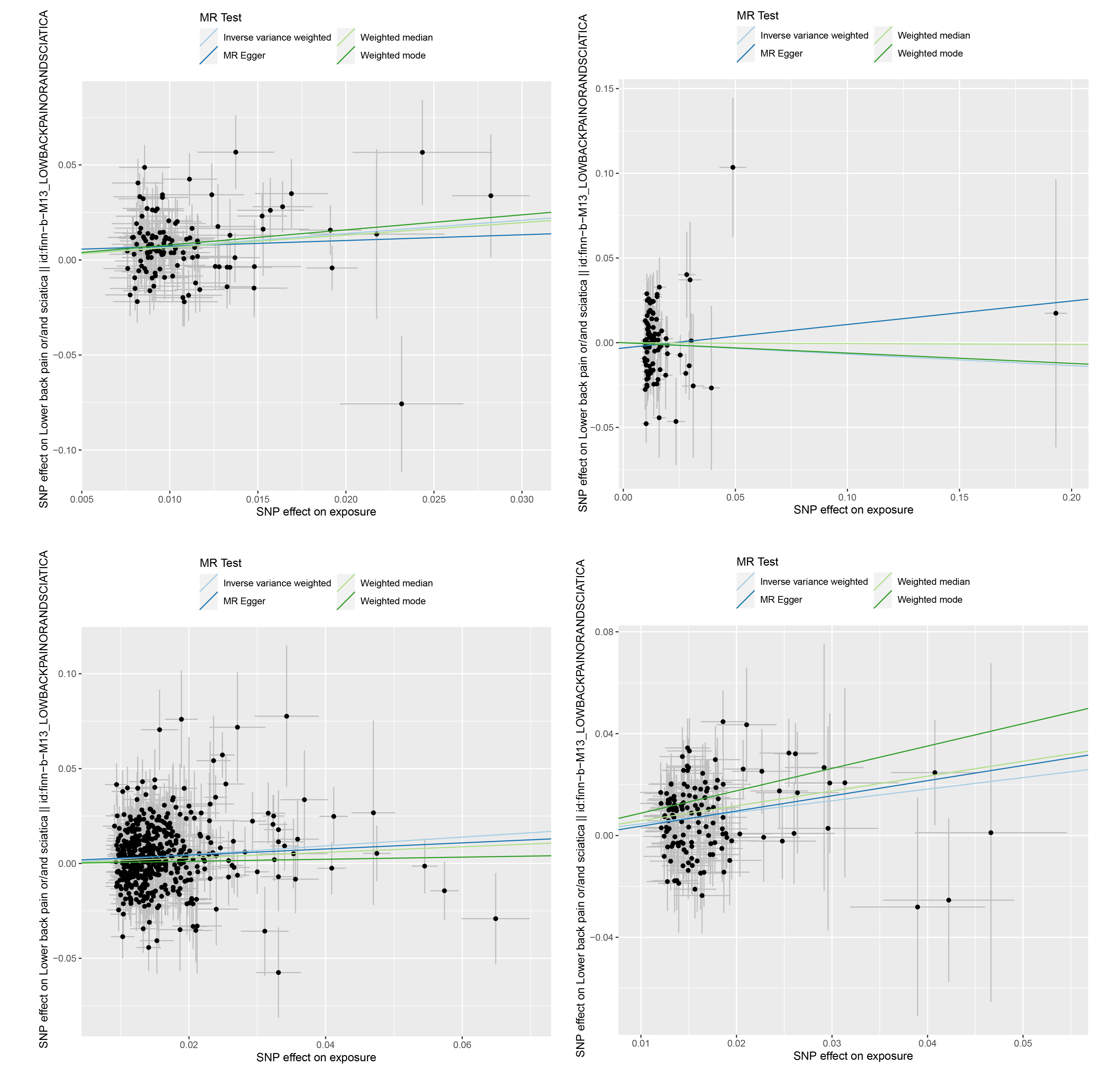


**D**

**C**

# Supplementary Figure S. Scatter plot of education on potential mediators.

A: Education on smoking index; B: Education on Alcohol Consumption; C: Education on BMI; D: Education on Leisure Television;

**A**

**D**

**C**

**B**


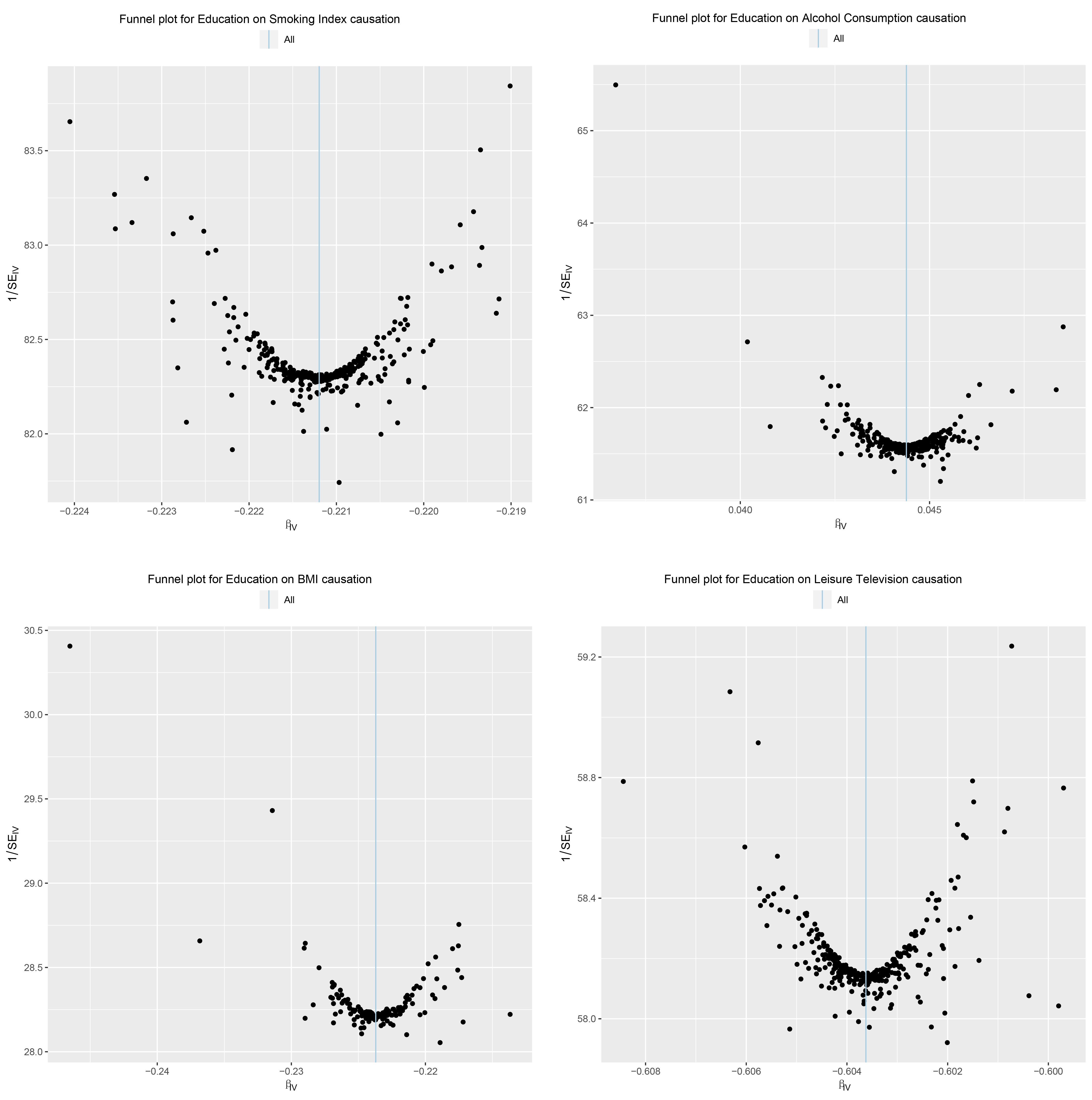


# Supplementary Figure S. Funnel plot of education on potential mediators.

A: Education on smoking index; B: Education on Alcohol Consumption; C: Education on BMI; D: Education on Leisure Television;

**A**

**D**

**C**

**B**


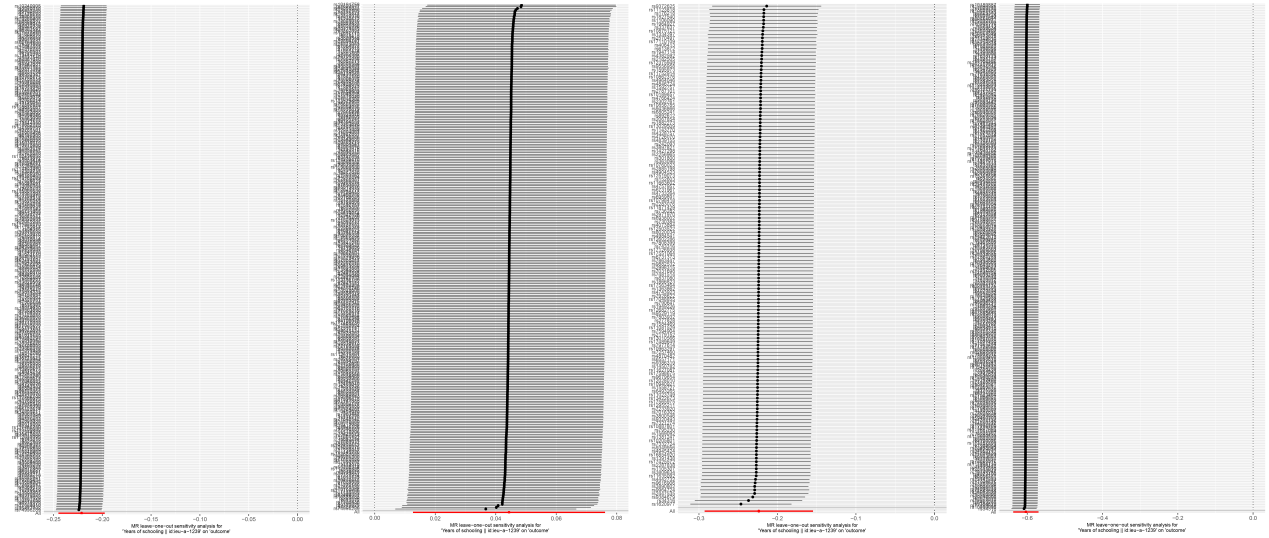


# Supplementary Figure S. Leave-one-out plot of education on potential mediators.

A: Education on smoking index; B: Education on Alcohol Consumption; C: Education on BMI; D: Education on Leisure Television;

**A**

**D**

**C**

**B**


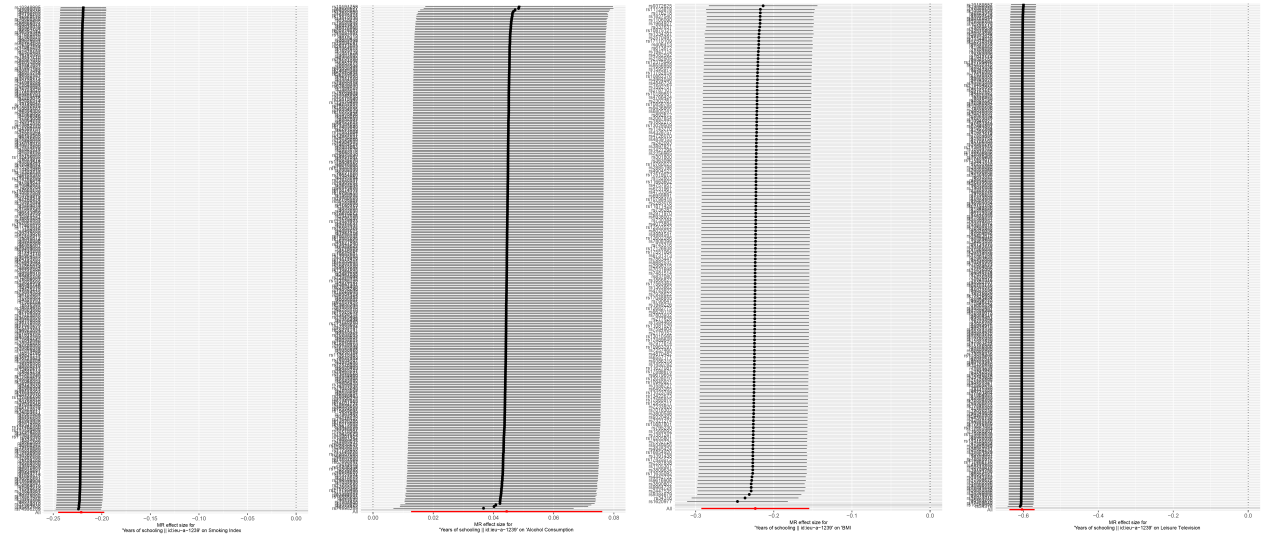


# Supplementary Figure S. Forest map of education on potential mediators.

A: Education on smoking index; B: Education on Alcohol Consumption; C: Education on BMI; D: Education on Leisure Television;


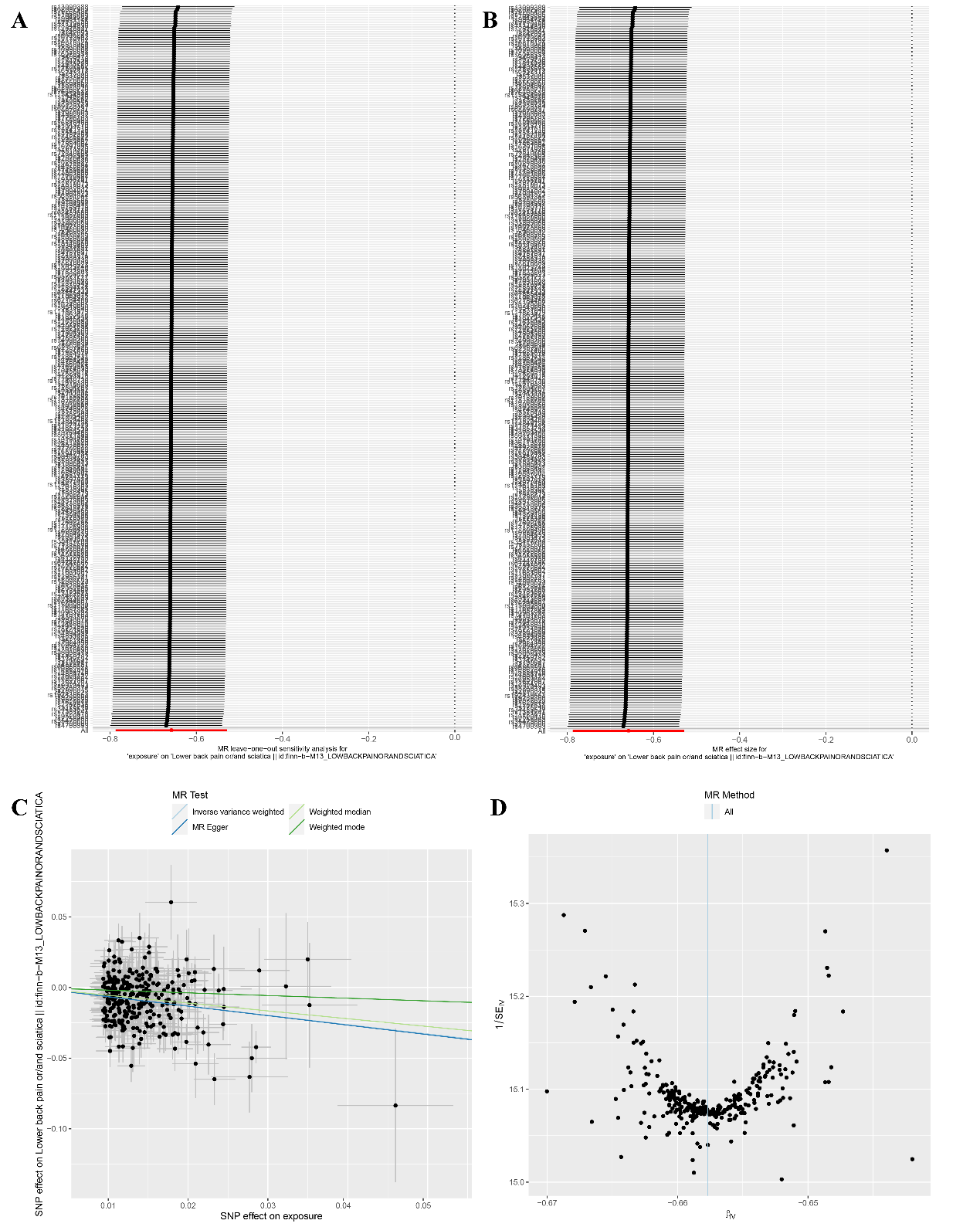


# Supplementary Figure S. Sensitivity analysis of education to LBP.

A: leave-one-out analysis plot; B: Forest map; C: Scatter plot; D: Funnel plot.

**References**

1. Cai D, Chen J, Wu Y, Jiang C. No causal association between tea consumption and 7 cardiovascular disorders: A two-sample Mendelian randomization study. *Front Genet* (2022) 13:989772. doi: 10.3389/fgene.2022.989772

2. Chu AY, Deng X, Fisher VA, Drong A, Zhang Y, Feitosa MF, et al. Multiethnic genome-wide meta-analysis of ectopic fat depots identifies loci associated with adipocyte development and differentiation. *Nat Genet* (2017) 49:125–130. doi: 10.1038/ng.3738

3. Okbay A, Beauchamp JP, Fontana MA, Lee JJ, Pers TH, Rietveld CA, et al. Genome-wide association study identifies 74 loci associated with educational attainment. *Nature* (2016) 533:539–542. doi: 10.1038/nature17671

4. Burgess S, Davies NM, Thompson SG. Bias due to participant overlap in two-sample Mendelian randomization. *Genet Epidemiol* (2016) 40:597–608. doi: 10.1002/gepi.21998
